# Proofreading experimentally assigned stereochemistry through Q2MM predictions in Pd-catalyzed allylic aminations

**DOI:** 10.1038/s41467-021-27065-2

**Published:** 2021-11-18

**Authors:** Jessica Wahlers, Jèssica Margalef, Eric Hansen, Armita Bayesteh, Paul Helquist, Montserrat Diéguez, Oscar Pàmies, Olaf Wiest, Per-Ola Norrby

**Affiliations:** 1 Department of Chemistry and Biochemistry, University of Notre Dame, Notre Dame, IN 46556 USA; 2 Departament de Química Física i Inorgànica, Universitat Rovira I Virgili, C/Marcel·li Domingo, 43007 Tarragona, Spain; 3 Oral Product Development, Pharmaceutical Technology & Development, Operations, AstraZeneca, Gothenburg, Sweden; 4 Data Science and Modelling, Pharmaceutical Sciences, R&D, AstraZeneca Gothenburg, Pepparedsleden 1, SE-431 83, Mölndal, Sweden; 5 Department of Chemistry and Molecular Biology, University of Gothenburg, Gothenburg, Sweden

**Keywords:** Reaction mechanisms, Asymmetric catalysis, Stereochemistry, Computational chemistry

## Abstract

The palladium-catalyzed enantioselective allylic substitution by carbon or nitrogen nucleophiles is a key transformation that is particularly useful for the synthesis of bioactive compounds. Unfortunately, the selection of a suitable ligand/substrate combination often requires significant screening effort. Here, we show that a transition state force field (TSFF) derived by the quantum-guided molecular mechanics (Q2MM) method can be used to rapidly screen ligand/substrate combinations. Testing of this method on 77 literature reactions revealed several cases where the computationally predicted major enantiomer differed from the one reported. Interestingly, experimental follow-up led to a reassignment of the experimentally observed configuration. This result demonstrates the power of mechanistically based methods to predict and, where necessary, correct the stereochemical outcome.

## Abstract (web summary)

A predictive model has been created for a stereoselective palladium-catalysed allylic amination reaction. Derived only from quantum chemical data, the method is accurate enough to reveal multiple erroneous assignments in literature experiments.

## Introduction

Computational chemistry has long promised the development of predictive methods in order to reduce the time needed to develop and optimize the conditions of reactions^1^. This has become especially desirable for predicting stereoselectivity in asymmetric catalysis because the identification of a chiral catalyst that gives high enantiomeric excess (*ee*) for a given substrate requires significant effort. While high-throughput experimentation allows for many different reaction conditions to be tested at once, this method still remains costly, especially for testing many different ligands^2,3^. Computational methods can not only predict which ligands would give the best results, reducing the time and cost needed to find the best catalyst^4^, but also give insight into the steric and electronic interactions that promote high stereoselectivity. Given the small energy differences involved, the computational methods need to be highly accurate while being fast enough to be useful for the synthetic chemist.

**Fig. 1:**
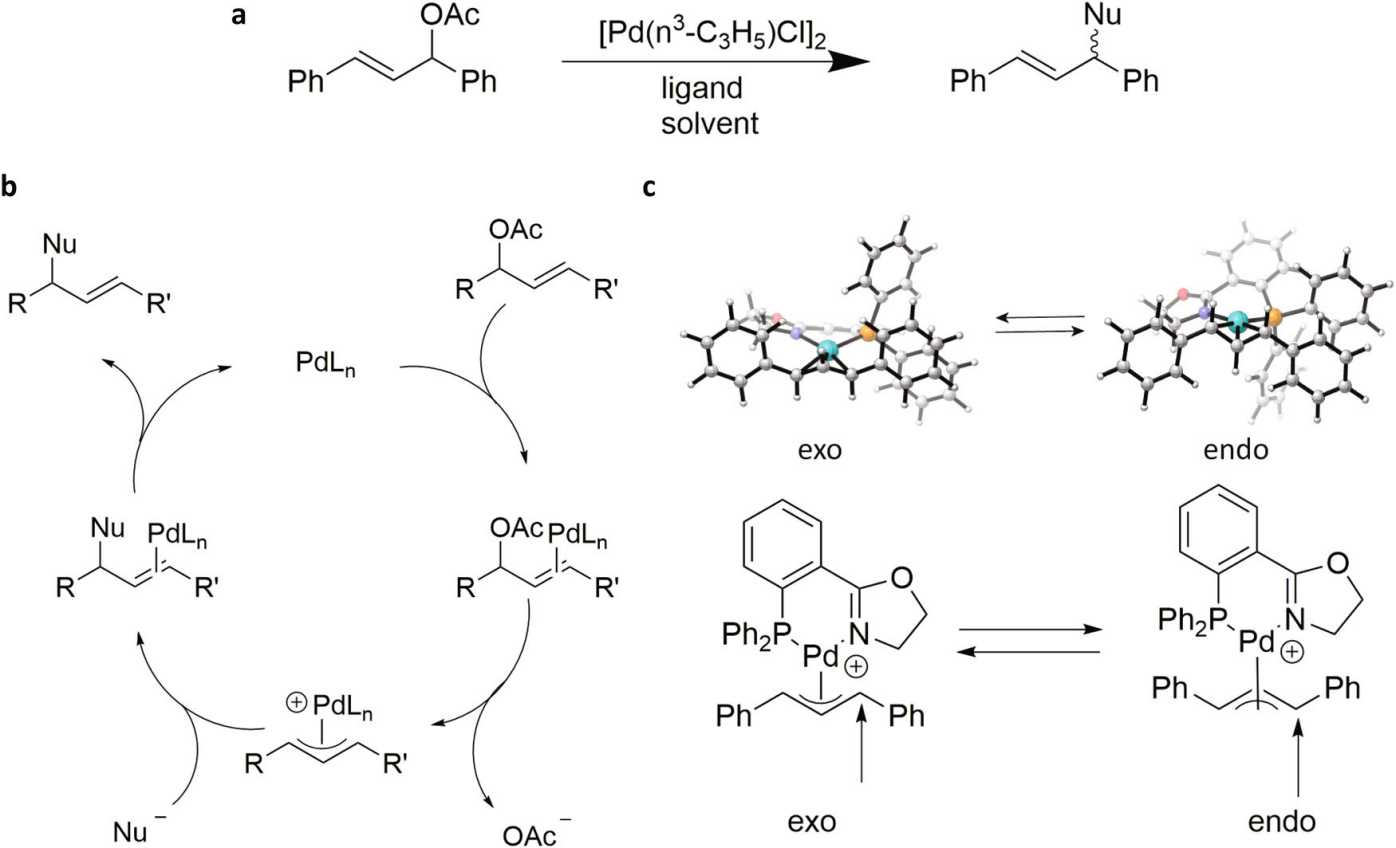
Pd-catalyzed allylic amination reaction. **a** Reaction modeled for the TSFF being developed. **b** Simplified mechanism of the reaction. **c** Exo-endo isomerization of the allyl.

A reaction of wide use in the pharmaceutical industry is the palladium-catalyzed asymmetric allylic substitution due to its mild conditions and ability to stereoselectively form a bond to carbon with a wide range of nucleophiles (Fig. 1a)^5–7^. Of particular interest is the allylic amination reaction, which forms a bond between a chiral carbon and an amine nitrogen. About 84% of pharmaceuticals contain at least one nitrogen atom, many of which are at a chirality center for which absolute configuration is important for desired therapeutic properties^8,9^. While this substitution reaction has been widely studied to determine the scope and mechanism, new substrates or nucleophiles usually require a new ligand screen to find the optimal catalyst^5,7,10–12^. The selectivity in this reaction depends on a complex interplay between steric interactions favoring a certain allyl geometry, dynamic interconversion through *exo-endo* isomerization of the allyl moiety, and electronic effects whereby the ligand can influence the regioselectivity of nucleophilic attack^7,13^.

The catalytic cycle of this reaction proceeds^7,14–16^ through an oxidative addition to form the reactive *η*^3^-allyl palladium intermediate, which has been studied by X-ray crystallography. (Fig. 1b). The *exo* and *endo* isomers of the Pd-allyl species are generally in rapid equilibrium with each other^13^. The nucleophile then attacks the allyl group in the stereoselectivity determining transition state. The most common chiral ligands to introduce stereoselectivity in this step are phosphorus and nitrogen based bidentate ligands^7,17,18^. There has been interest in using P,N ligands because they can discriminate between the two terminal allylic carbons based on their electronic differentiation, directing the nucleophile towards the allylic carbons trans to the phosphorus atom. Some common ligands used for this reaction include the PHOX ligands, phosphite-oxazoline ligands, and aminoalkyl-phosphine ligands^7,18–22^. These ligands can control *exo-endo* preference through the chiral oxazoline/amine moiety which, thanks to the trans phosphorus, is in close proximity to the reacting allyl terminus (Fig. 1c)^17^.

There have been a few methods developed to predict stereoselectivity in asymmetric catalysis. Calculation of the transition state structures and the energy difference between the structures leading to the R and S enantiomers by DFT^14,16,23^ is slow and typically does not sample a sufficiently large number of conformations. Another method is to predict stereoselectivity by fitting to various steric and electronic parameters^24^. Recently, there has been a push to use machine learning methods, but these methods often need large data sets of high quality to train the model, and offer limited insight into details of the reaction mechanisms and which parameters contribute to high stereoselectivity^25^.

Quantum Guided Molecular Mechanics (Q2MM) was developed to predict stereoselectivity, combining the speed of molecular mechanics (MM) with the accuracy of DFT^26–29^. It uses transition state force fields (TSFFs) that are trained on electronic structure calculations of simplified models of the stereoselecting transition state. Because no empirical data are used to fit the force field, the results are true predictions. Once a force field has been developed, it can be used to perform a Monte-Carlo conformational search to determine the Boltzmann-averaged energy difference between the transition state structures that lead to the R and S enantiomers. CatVS is a program that automates the process of building full TS structures as well as adding conformational search parameters to the full system^30^. These energy differences are then compared and validated by the experimental results.

A ground state force field of the reactive intermediate for this reaction was previously developed to study steric interactions that contribute most to the stereoselectivity of the reaction^31–33^. However, predictions using the ground state force field requires manual inspection of geometries and assumptions about preferred nucleophilic attack vectors. For the rapid screening of new ligands, substrates, and nucleophiles, a TSFF is better suited to predict stereoselectivity, since it is the difference in transition state energies rather than ground states that govern preference for formation of a particular stereoisomer of the major product. Computational insight could also elucidate which interactions influence selectivity to find the optimal ligand for a given substrate and nucleophile.

Here, we describe the development of a TSFF for the palladium-catalyzed allylic amination reaction to predict stereoselectivity as well as understand the interactions in the transition state that lead to higher selectivity. Numerous discrepancies between the predicted and literature values are re-investigated experimentally, and found to be due to misassigned absolute stereochemistry in the literature.

## Results and discussion

A training set consisting of 21 simplified TS structures (see Supplementary Fig. 1 and Supplementary Table 1) that capture the steric and electronic information around the reaction coordinate and metal center was used to parameterize the TSFF. In addition, one structure representing a full ligand (achiral) and a full allyl structure was included to ensure that the interactions being parameterized accurately describe the steric and electronic interactions as well as capture the geometry of a full system. The reference structures were optimized using M06-D3/LANL2DZ/6-31 + G* (for details see Methods section), and the TSFF was parameterized by Q2MM as described earlier^26,27^. Internal validation of the optimized parameters such as structural data and Hessian eigenvalues between the QM and MM optimized transition structures is shown in the Supplementary Information. Minor deviations in the bond length of the forming bond between the allylic carbon and the amine are observed for cases with sterically bulky ligands where the forming bond is usually shorter. No significant deviations between QM and MM in the angles and torsions of the training set are observed. Overall, the *R*^2^ values for the internal validation ranges from 0.988 to 0.998 for geometric and Hessian eigenvalues, respectively, and 0.822 for charges, which are typical values for internal validations of TSFFs^28,34,35^.

The next step is the external validation by prediction of selectivities for ligand-substrate combinations from the literature that are not part of the training set. Using CatVS^30^, the libraries of TS structures can rapidly and automatically be prepared for conformational searches by merging substrate, ligand, and nucleophile sub-libraries onto a template. The calculation of each pair of diastereomeric transition states takes between 15 and 60 min on a single core, making this method suitable for high-throughput calculations on even a modest cluster. The output is given as differences in TS energies for forming the two enantiomeric products, and also as enantiomeric ratio and excess, calculated from Eq. 1. For cases with more than two competing transition states, the ratio is obtained by a Boltzmann summation over diastereomeric pathways where positive values indicate a preference of the S enantiomer:

1 $$\begin{array}{c}{{{{{\rm{enantiomeric}}}}}}\,{{{{{\rm{ratio}}}}}}:\,er={e}^{\Delta \Delta {G}^{{{\ddagger}} }/RT}\\ {{{{{\rm{enantiomeric}}}}}}\,{{{{{\rm{excess}}}}}}:\,ee=100 \% \frac{er\,-\,1}{er\,+\,1}\end{array}$$

**Fig. 2:**
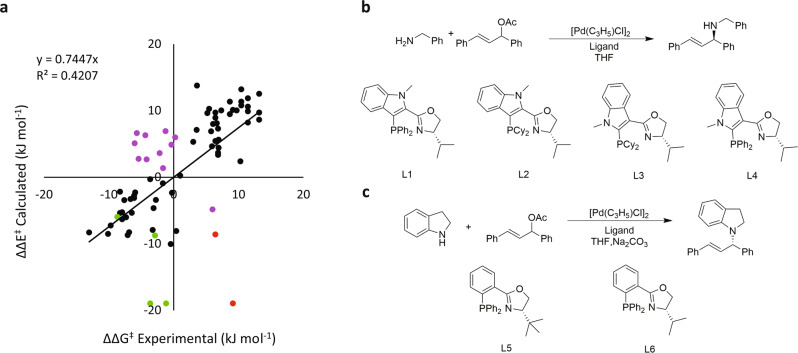
Comparison of relative energies of the experimental values to the calculated MM values. **a** The largest systematic errors in the TSFF are for ligands containing an indole backbone (green), examples of predicting opposite absolute configuration with a PHOX ligand (red), and examples of predicting opposite absolute configuration with a phosphite-oxazole ligand (purple). **b** Reactions that are catalyzed by ligands with an indole backbone (green data points). **c** Reaction of the two examples that give the opposite absolute configuration when catalyzed by the PHOX ligands (red data points).

A validation dataset containing 77 structures (Supplementary Figs. 3 and 4, and Supplementary Table 3) assembled from the literature^19,21,36–43^ was used to test the performance of the TSFF for systems different than the training set (Fig. 2a). 1,3-Diphenyl propenyl was used as the allyl component reacting with 16 different amines, catalyzed by the Pd-complexes of 53 different P,N ligands. Most ligands, including PHOX and norbornyl ligands as well as ligands with different substituents on the nitrogen are well described by the force field. The experimental free energy differences between ensembles leading to the enantiomeric product, ΔΔG^‡^, was derived from Eq. 2 where positive ΔΔG^≠^ indicate that the S enantiomer is the preferred product

2 $$\Delta \Delta {G}^{{{\ddagger}} }=RT\,{{{{\mathrm{ln}}}}}(er)\qquad er=\frac{100 \% +ee}{100 \% -ee}$$

The final test showed larger deviations than are usually seen with Q2MM. The mean unsigned error (MUE) over the 77 cases was 4.4 kJ/mol and the *R*^2^ value only 0.41 (Fig. 2). Although these value are not as good as those of several published TSFFs^26,27^, it is clear from Fig. 2a that the vast majority of cases in the validation set is reproduced well and that the deviation are due to a small number (<20%) of cases with significant differences between the computed and experimental results.

Historically, the path to systematic improvements of force fields is through the detailed analysis of the outliers^44^. Such an analysis for the results in Supplementary Table 3 indicates that the high MUE originates from a few systematic deviations that are color-coded in Fig. 2a. The first set of ligands where the predictions deviate from the experimental results are IndPHOX ligands, shown in green in Fig. 2a. Experimentally, L1 and L4 give very different selectivities of 52 % ee and 94 % ee, respectively^43^. Sterically, the ligands are very similar, and thus the force field predicts that these two ligands should give similar selectivity results with L1 giving 93.5 % ee and L4 giving 95.3 % ee. Similar results are obtained for the related ligands L2 and L3, where the selectivities are predicted to be too high. In L1 and L2, the phosphorus is connected to the very electron-rich 3-position of the indole. It is plausible that the resulting catalytic activity is so high that the nucleophilic attack is faster than the exo-endo isomerization. The Q2MM model depends on a Curtin-Hammett situation where the exo and endo isomers are in rapid equilibrium. If this effect is negated by a too fast nucleophilic attack, the reaction becomes stereospecific, and a racemic allylic acetate will in such a situation yield low selectivity. Thus, this seems to be a case of a change in mechanism for which the Q2MM-derived TSFF is therefore not applicable.

More interesting are cases where the predicted stereoselectivity is high but opposite to the one reported in the literature. These include two examples of PHOX ligands (L5 and L6 in Fig. 2c) shown in red in Fig. 2a^39^ and a series of reactions using a phosphite-oxazole ligand shown in purple in Fig. 2a and discussed below. The force field predicts that the absolute product configuration should be *R* for the two PHOX ligands while the experimental results has *S* as the absolute stereochemistry. While the absolute configuration was determined by a crystal structure, the reported Flack parameter was 0.1 with an uncertainity of 0.6. This large degree of uncertainty could suggest that the absolute stereochemistry was reported incorrectly. L6 has previously been used by another group with similar reaction conditions, but using benzylamine rather than indoline as the nucleophile^21^. In that case, the absolute configuration predicted by the force field matches the absolute configuration described in the literature which was reported as *R*. A substrate screen performed by Liu and coworkers using a d-camphor-based chiral P,N ligand did not show a change in the absolute stereochemistry between the benzylamine and indoline nucleophiles^37^. This suggest that there should not have been a change in the absolute stereochemistry when using L6 for the benzylamine and indoline nucleophiles that was seen experimentally. To study this, the stereochemistry assignment was reexplored experimentally (see Supplementary Information). Comparison of the chromatographic eluting order and the polarimetric analysis of the aminated product using ligand **L5** with the literature indicated that the major enantiomer formed is the (*R*)-(-)-1-(1,3-diphenylallyl)indoline as predicted by the calculations.

**Fig. 3:**
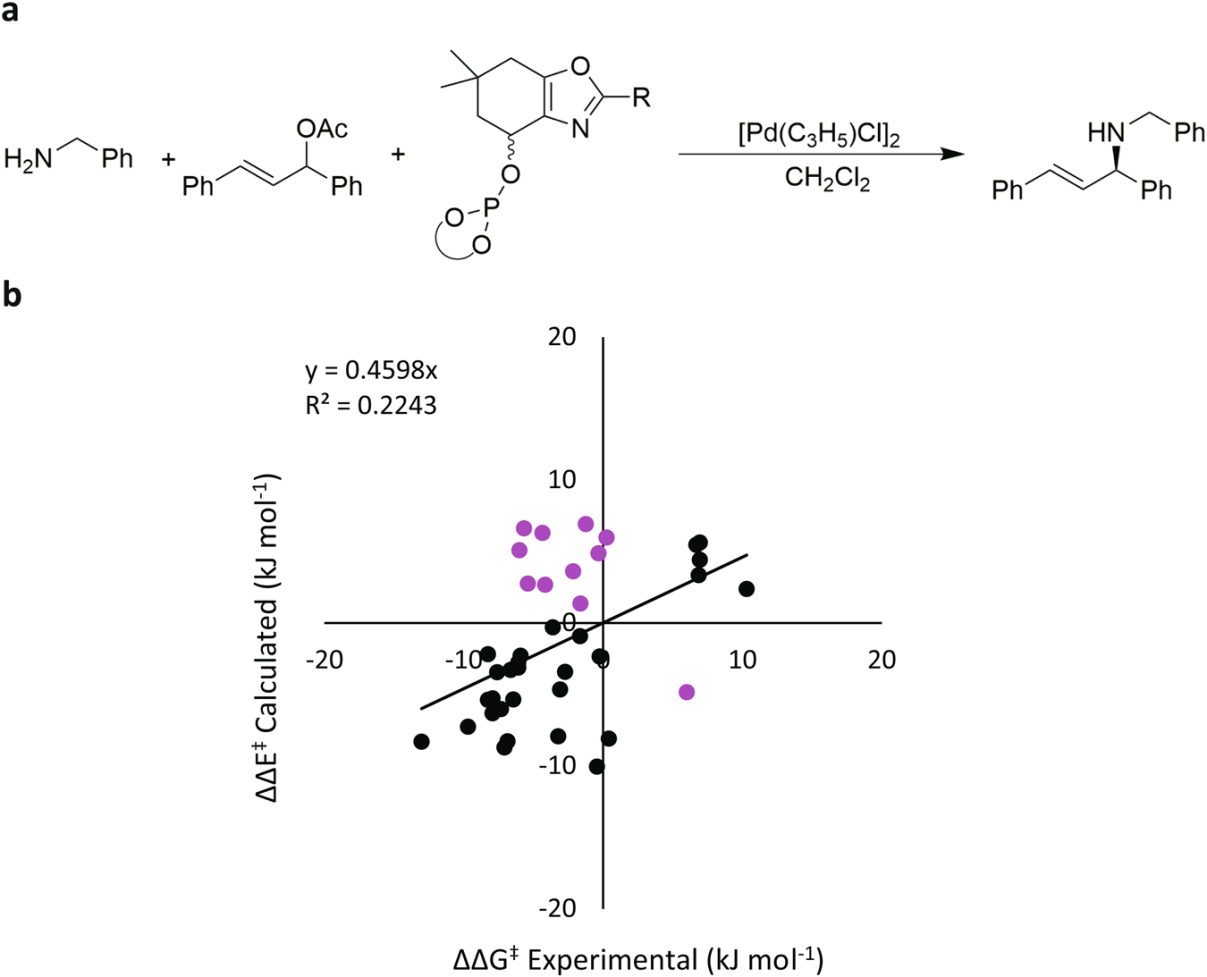
Comparison of relative energies of the experimental values to the calculated MM values for 39 phosphite-oxazole ligands. **a** Reaction corresponding to the 11 mismatched data points. **b** Calculated vs. experimental stereoselectivity with mismatched cases in purple.

The possibility for the mismatch between computed and reported absolute stereochemistry was also explored for the phosphite-oxazole ligands (Fig. 3b) for which a larger dataset is available. In total, 39 different ligand-substrate combinations for this reaction were studied^36,37^, 11 of which showed the mismatch (Fig. 3a). Specifically, the TSFF predicts that the absolute configuration to be S while the literature reports an absolute configuration of R for the products. An analysis of the 28 cases where the predicted and reported stereochemistry match (black in Fig. 3a) did not show any significant differences to the 11 cases that did.

We therefore initiated experimental studies to check the original stereochemical assignment. For that purpose, we reexamined several of the mismatched phosphite-oxazole ligands in allylic amination of (*rac*)-1,3-diphenyl allyl acetate with benzylamine (see Supplementary Table 5). In all cases, chromatographic comparison of the aminated product to known samples revealed that the original assignment in the literature was incorrect, and that the dominant stereoisomer was the one predicted by the Q2MM force field. This shows that the predictions of the model in this case are qualitatively and quantitatively correct even when they contradict assignments of the absolute stereochemistry in the literature.

**Fig. 4:**
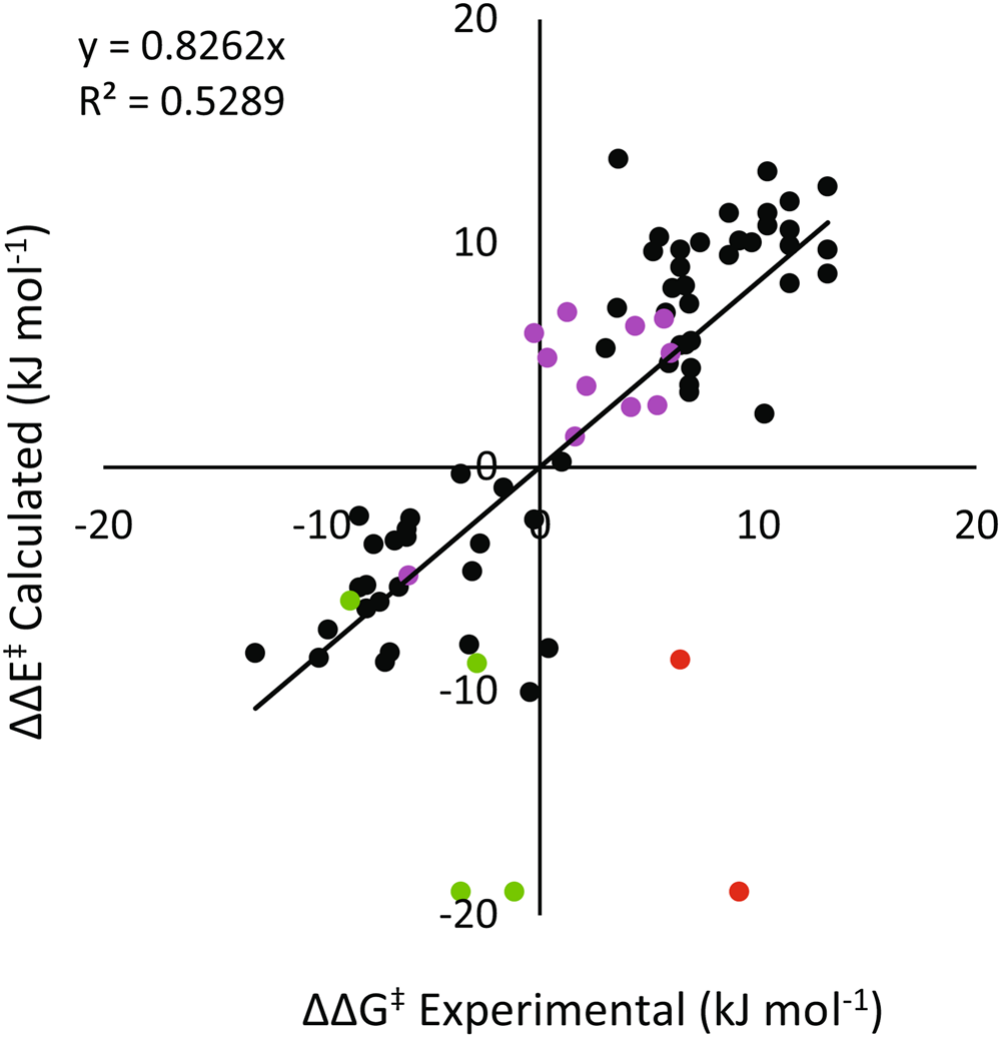
Comparison of relative energies of the corrected experimental values to the calculated MM values. The absolute configurations for the 11 data points in purple have been corrected.

Having experimentally confirmed that the computationally predicted absolute stereochemistry is correct, the overall MUE over 77 cases decreased to 3.2 kJ/mol (Fig. 4). This value is still affected by the a small number of data points where we believe a mechanistic shift has invalidated the Q2MM model as discussed earlier. Excluding the IndPHOX results (green dots) as being out of scope due to change in mechanism the remaining 95% of the 77 cases are predicted by the TSFF with an MUE of 2.8 kJ/mol and an *R*^2^ of 0.72, which is typical Q2MM-derived force fields^26,27^.

To conclude, mechanism-based prediction of using Q2MM-derived TSFF has shown an ability not only to predict reaction outcome in advance of experimental work but also to correct stereochemical assignments of sets of reported data. We note that other methods that are based on machine learning (ML), which are becoming rapidly popular in the field, are highly sensitive to such errors in input data and are unable to correct for such systematic errors for more than a very small percentage of the training data. Instead, the highly complex correlation models obtained from many ML methods will attempt to fit the spurious data, resulting in models that give erroneous assignments for sets within the applicability domain. Although it is obvious that stringent quality control of the training for ML models is essential, this is in practice difficult to do for large datasets from the literature. We thus believe that fast TSFF calculations, which describe the underlying causes for the observed selectivities through physically meaningful parameters, provide a new, high-throughput tool to “proofread” stereochemical assignments that could be highly useful for researchers engaged in studies of asymmetric synthesis.

## Methods

DFT calculations of the training set were performed in the gas phase using Gaussian^45^. The M06^46^ functional form was used with a D3 empirical dispersion correction^47^. The basis sets used were LANL2DZ for palladium and 6-31 + G* for all other atoms. CHELPG^48^ with a vdW radius of 2.4 Å for palladium was used to calculate the partial charges. Frequency analysis confirmed that the transition state structures contained one negative vibration corresponding to the formation of the carbon–nitrogen bond.

The TSFF parameters for the atoms involved in bond formation (see Supplementary Information) were fit and optimized using the Q2MM method. The MM3* force field^49^ was used as the functional form of the TSFF and for any parameter that were not being fit. The full TS systems were automatically generated by CatVS and subjected to 40.000 steps of Monte Carlo conformational search using the mixed torsional/low-mode sampling in Macromodel^50^ with a constant dielectric of 1.0. The resulting conformations of the diastereomeric transition states were, after Boltzmann averaging, used for prediction of selectivity^27^.

## Supplementary information

Supplementary Information

## Data Availability

An open-source version of the Q2MM/CatVS code, together with a library of the currently available TSFFs, reaction templates and ligand libraries, is available to the scientific community free of charge as part of the Q2MM package for the generation of TSFFs in the GitHub repository (https://github.com/Q2MM/q2mm).

## Data availability

All other data are available from the authors upon reasonable request.
