# Supplementary material for: Proofreading experimentally assigned stereochemistry through Q2MM predictions in Pd-catalyzed allylic aminations
Source: Nat Commun. 2021 Nov 18;12:6719. doi: 10.1038/s41467-021-27065-2 (PMC8602308; doi:10.1038/s41467-021-27065-2)
Supplement: Supplementary file 1 — Supplementary Information [file 41467_2021_27065_MOESM1_ESM.pdf]

Supplementary Information

Proofreading Experimentally Assigned Stereochemistry Through Q2MM Predictions in Pd-Catalyzed Allylic Aminations

J. Wahlers et al.

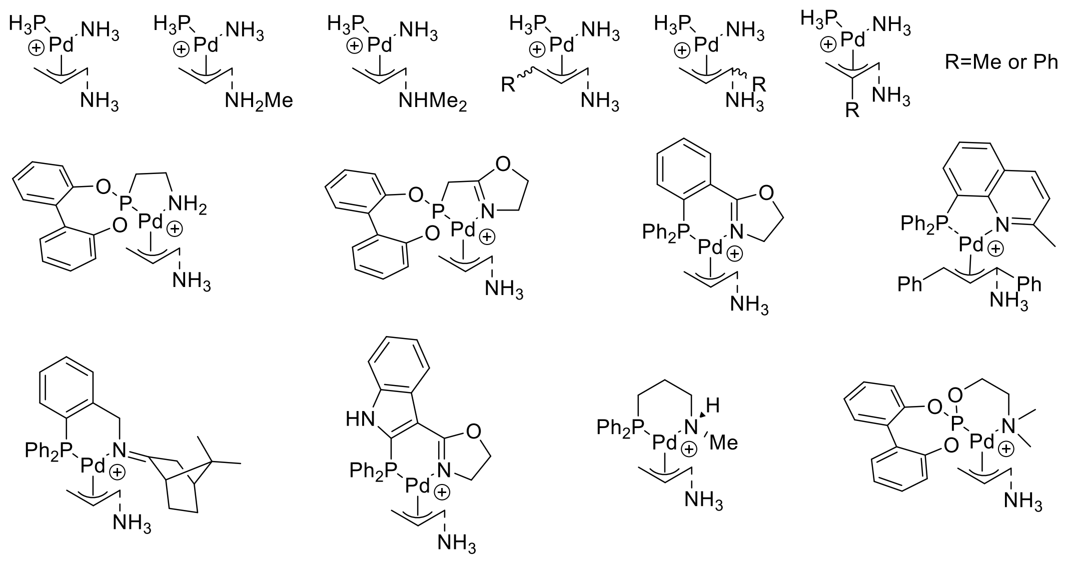

Supplementary Figure 1. Training set structures used to fit the force field parameters

Supplementary Table 1. Coordinates for the DFT optimized structures in the training set

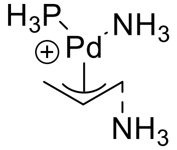

TS 1.
Gibbs Free Energy: -699.798031
Imaginary Frequency: -333.18
Cartesian coordinates and point charges:

Pd	-0.43973	0.161508	0.102093	-0.17746
P	-2.5037	-0.94055	-0.30086	0.28649
H	-2.9894	-1.81422	0.692499	0.01425
H	-3.71257	-0.2516	-0.54564	0.0121
H	-2.5784	-1.83259	-1.38997	0.00364
N	-1.12861	2.324886	-0.01298	-0.64035
H	-1.55715	2.543185	-0.91141	0.28803
H	-1.83373	2.502176	0.701135	0.3054
H	-0.3901	3.010267	0.133721	0.28832
C	1.608243	-0.34529	0.693343	-0.03142
C	2.280925	0.411347	-0.2982	0.07537
C	0.832858	-1.4884	0.353728	-0.34887
H	2.566501	1.439726	-0.08438	0.0946

H	0.519335	-2.17281	1.142224	0.16335
H	2.070699	0.202237	-1.34705	0.09405
H	1.770133	-0.08597	1.740897	0.10638
N	4.152029	-0.22187	-0.40967	-0.5298
H	4.121833	-1.22629	-0.58902	0.27494
H	4.729114	0.217807	-1.12916	0.29212
H	4.605498	-0.08233	0.494163	0.27382
H	0.965259	-1.95778	-0.62608	0.15502

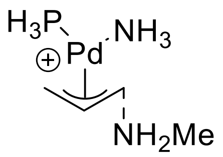

TS 2.
Gibbs Free Energy: -739.045407
Imaginary Frequency: -257.33
Cartesian coordinates and point charges:

Pd	0.795218	0.151145	-0.13183	-0.12461
P	2.873176	-0.77132	0.547701	0.23545
H	3.503667	-1.67004	-0.33565	0.02497
H	4.004071	0.017403	0.853521	0.02616
H	2.895393	-1.58427	1.698597	0.01675
N	1.305522	2.357937	-0.09612	-0.60022
H	1.431967	2.70453	0.853903	0.27696
H	2.176663	2.537285	-0.59371	0.28987
H	0.598434	2.946984	-0.53215	0.28433
C	-1.11037	-0.60051	-0.93203	-0.05376
C	-1.88922	0.242562	-0.11774	-0.16231
C	-0.30235	-1.62808	-0.37249	-0.27988
H	-2.23212	1.205658	-0.49124	0.15718
H	0.155982	-2.36284	-1.03449	0.14624
H	-1.83136	0.150562	0.966821	0.14265
H	-1.14806	-0.46399	-2.01365	0.12344
N	-3.86854	-0.44588	-0.07671	-0.19749
H	-3.8147	-1.43173	0.179738	0.21015
H	-4.22822	-0.40407	-1.03031	0.20713
H	-0.52397	-1.99808	0.633012	0.14486
C	-4.70035	0.318956	0.854749	-0.09178
H	-5.74178	-0.02494	0.884211	0.0761
H	-4.27813	0.237783	1.862495	0.07687
H	-4.69066	1.375	0.562255	0.07093

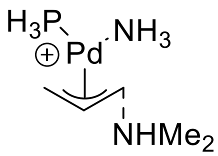

TS 3.
Gibbs Free Energy: -778.29767
Imaginary Frequency: -195.25
Cartesian coordinates and point charges:

Pd	1.070945	0.140616	-0.1082	-0.14318
P	3.211061	-0.72942	0.431691	0.25725
H	3.703296	-1.77835	-0.36961	0.02152
H	4.376135	0.068131	0.439088	0.02104
H	3.389561	-1.34104	1.688441	0.01915
N	1.591306	2.324256	-0.38814	-0.55815
H	1.953655	2.744459	0.466788	0.27289
H	2.311822	2.434345	-1.1004	0.27771
H	0.802401	2.896341	-0.68416	0.27175
C	-0.8955	-0.70025	-0.6302	0.05625
C	-1.53917	0.302162	0.110063	-0.21596
C	-0.04214	-1.64903	-0.00461	-0.33416
H	-1.91684	1.195846	-0.38445	0.15783
H	0.351189	-2.47849	-0.59212	0.16487
H	-1.40181	0.368672	1.188598	0.14717
H	-1.0291	-0.71893	-1.71298	0.10016
N	-3.61535	-0.25971	0.425236	-0.03392
H	-3.59021	-1.00748	1.118029	0.20131
H	-0.16698	-1.86322	1.060731	0.14966
C	-4.33893	0.895711	0.940758	-0.18694
H	-5.4063	0.686249	1.108315	0.09638
H	-3.89265	1.227804	1.885537	0.08143
H	-4.26524	1.715536	0.213412	0.08295
C	-4.13399	-0.76066	-0.84005	-0.098
H	-5.19371	-1.04942	-0.77555	0.07168
H	-4.03758	0.024656	-1.6025	0.07284
H	-3.55031	-1.6315	-1.16077	0.04646

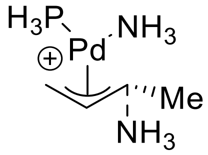

TS 4.
Gibbs Free Energy: -739.059094
Imaginary Frequency: -327.40
Cartesian coordinates and point charges:

Pd	0.628012	0.143881	-0.182874	-0.17942
N	1.300373	2.320854	-0.152549	-0.59957
H	1.956442	2.48129	-0.9159	0.29072
H	0.549007	2.997713	-0.271055	0.26959
H	1.785444	2.568888	0.708383	0.28221
P	2.657647	-0.933085	0.371025	0.2524
H	3.843572	-0.231616	0.686264	0.01621
H	2.673087	-1.814759	1.471445	0.00948
H	3.215544	-1.811894	-0.579629	0.01847
C	-1.407701	-0.353186	-0.823073	-0.23463
C	-2.217438	0.399305	0.077685	0.51092
C	-0.653305	-1.499893	-0.452351	-0.21942
H	-2.49967	1.394407	-0.272825	0.00738
H	-0.306054	-2.170976	-1.238469	0.13962
H	-0.820048	-1.991177	0.509536	0.09832
H	-1.493028	-0.097569	-1.881554	0.11982
N	-4.067638	-0.308069	-0.218476	-0.68304
H	-4.054203	-1.286994	0.072753	0.29731
H	-4.832243	0.161155	0.270787	0.32614
H	-4.26928	-0.287941	-1.218743	0.3039
C	-2.170779	0.243333	1.56763	-0.43518
H	-3.035788	0.715054	2.04668	0.13049
H	-2.12865	-0.808567	1.874915	0.15221
H	-1.271193	0.733904	1.962074	0.1261

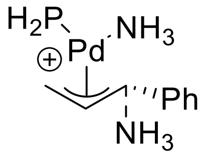

TS 5.
Gibbs Free Energy: -930.602498
Imaginary Frequency: -339.31
Cartesian coordinates and point charges:

Pd	1.380832	0.200234	0.197762	-0.24273
N	1.54318	-0.75283	2.267371	-0.56928
H	2.491174	-1.06337	2.473458	0.28843
H	1.268437	-0.13641	3.029804	0.26727
H	0.944299	-1.57595	2.314607	0.24977
Pd	2.99361	-1.05983	-0.99297	0.30405

H	3.808554	-2.04938	-0.39563	-0.0008
H	2.575914	-1.82548	-2.10155	-0.00845
H	4.027738	-0.35336	-1.6412	0.00722
C	0.036209	1.902879	-0.08588	-0.08851
C	-1.17657	1.408527	0.5087	0.2216
C	0.627484	1.46981	-1.30139	-0.28913
H	1.389894	2.105865	-1.75189	0.14909
H	0.0868	0.846118	-2.01361	0.11343
N	-2.53882	2.695058	0.084409	-0.72574
H	-2.6005	2.751946	-0.93332	0.30782
H	-3.4585	2.436571	0.448754	0.30909
H	-2.28865	3.620059	0.438579	0.35932
H	-1.23388	1.603201	1.583651	0.0692
C	-3.20937	-2.23525	-0.45694	-0.08546
C	-2.8848	-1.9248	0.861323	-0.04938
C	-2.23102	-0.73189	1.151602	-0.19739
C	-1.87297	0.155143	0.129099	0.16641
C	-2.22326	-0.15738	-1.1905	-0.15716
C	-2.88545	-1.34502	-1.47988	-0.08876
H	-3.72854	-3.16355	-0.68732	0.12194
H	-3.15278	-2.60675	1.666206	0.11332
H	-1.98961	-0.48243	2.186964	0.11058
H	-1.99134	0.533812	-2.00108	0.12586
H	-3.15402	-1.57592	-2.50907	0.1231
H	0.470475	2.7741	0.410857	0.09531

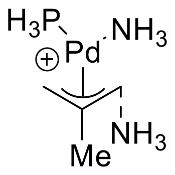

TS 6.
Gibbs Free Energy: -739.057457
Imaginary Frequency: -358.98
Cartesian coordinates and point charges:

Pd	-0.5713	0.151886	-0.01702	-0.23428
N	-1.20329	2.34163	0.015895	-0.62788
H	-1.92544	2.487632	0.719714	0.29977
H	-0.45358	2.995884	0.231767	0.28355
H	-1.59908	2.632022	-0.87713	0.28392
P	-2.70466	-0.88835	-0.21492	0.33437
H	-3.93228	-0.19838	-0.09197	-0.0048
H	-3.0107	-1.58237	-1.40378	-0.0067

H	-2.99929	-1.93205	0.686142	0.00082
C	1.514618	-0.38754	0.392367	0.30403
C	2.085219	0.397488	-0.65438	0.00568
C	0.716889	-1.51425	0.046666	-0.49801
H	2.343718	1.436659	-0.44498	0.09423
H	0.468331	-2.24599	0.81749	0.18928
H	1.764847	0.206739	-1.67864	0.09605
H	0.757435	-1.92244	-0.96741	0.17111
N	3.908982	-0.15994	-0.91447	-0.53342
H	3.908203	-1.17184	-1.05234	0.27533
H	4.367816	0.276584	-1.71642	0.30311
H	4.461927	0.043975	-0.08065	0.28406
C	1.945354	-0.12524	1.81123	-0.29553
H	1.216564	-0.52016	2.52868	0.11113
H	2.907997	-0.61133	2.03984	0.0768
H	2.060717	0.949042	2.011183	0.08736

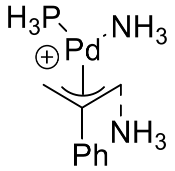

TS 7.
Gibbs Free Energy: -930.609145
Imaginary Frequency: -267.14
Cartesian coordinates and point charges:

Pd	-1.39357	0.07256	0.144667	-0.30152
N	-1.93811	-0.14181	2.335033	-0.47469
H	-1.57939	-1.02911	2.686941	0.25326
H	-1.5567	0.588102	2.934675	0.24554
H	-2.94467	-0.14819	2.491523	0.26185
P	-3.18039	-1.10233	-0.88804	0.36403
H	-4.25393	-1.67141	-0.16798	-0.00308
H	-3.94259	-0.43361	-1.86673	-0.00299
H	-2.84165	-2.23891	-1.6486	-0.00213
C	0.594038	0.799998	-0.47949	0.06308
C	0.39909	1.939718	0.330224	0.05356
C	-0.29248	0.599818	-1.57828	-0.3233
H	-0.10224	-0.22178	-2.26989	0.15046
H	-0.39258	2.648029	0.095918	0.1092
H	-0.81502	1.455269	-2.0146	0.15562

N	1.921661	3.263565	-0.22754	-0.59918
H	1.819391	3.466248	-1.22194	0.27453
H	2.008508	4.15381	0.264382	0.30064
H	2.799492	2.755535	-0.11052	0.22094
H	0.78367	1.96972	1.346277	0.08879
C	3.956238	-1.75823	0.267985	-0.09716
C	3.192428	-1.28839	1.3358	-0.05645
C	2.090568	-0.47431	1.100301	-0.21486
C	1.732131	-0.11462	-0.20599	0.21524
C	2.506785	-0.59125	-1.26915	-0.18932
C	3.609484	-1.40906	-1.03395	-0.05415
H	4.817824	-2.39682	0.452829	0.1149
H	3.45232	-1.56533	2.35613	0.1059
H	1.486309	-0.13438	1.944169	0.12215
H	2.254079	-0.30635	-2.29093	0.10688
H	4.202953	-1.768	-1.87294	0.11225

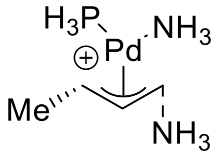

TS 8.
Gibbs Free Energy: -739.056054
Imaginary Frequency: -345.50
Cartesian coordinates and point charges:

Pd	-0.54154	0.305638	0.115982	-0.23144
N	-1.373	2.385541	-0.32489	-0.58724
H	-1.81584	2.432724	-1.24128	0.2718
H	-2.08695	2.628085	0.360688	0.29364
H	-0.67981	3.130619	-0.29555	0.27024
P	-2.51981	-0.98251	-0.13125	0.27405
H	-3.72075	-0.47193	-0.67389	0.01313
H	-2.46089	-2.15795	-0.9107	0.00219
H	-3.08046	-1.56026	1.02681	0.01103
C	1.522169	0.032331	0.793172	-0.264
C	2.190232	0.732818	-0.24306	0.26401
C	0.853826	-1.21463	0.598205	0.0788
H	2.390139	1.794304	-0.10551	0.05683
H	0.544637	-1.72069	1.517156	0.06518
H	2.032475	0.439747	-1.27991	0.04147
H	1.602571	0.433994	1.805044	0.12352
N	4.075207	0.221116	-0.24203	-0.56412

H	4.123287	-0.78751	-0.39338	0.28185
H	4.659582	0.680663	-0.94335	0.29593
H	4.466418	0.415618	0.680629	0.28519
C	1.133376	-2.14774	-0.5483	-0.22163
H	0.2828	-2.81466	-0.73391	0.08763
H	1.345668	-1.62829	-1.49181	0.07167
H	1.992208	-2.79953	-0.31922	0.0803

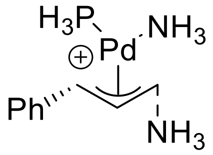

TS 9.
Gibbs Free Energy: -930.605574
Imaginary Frequency: -321.61
Cartesian coordinates and point charges:

Pd	1.499123	0.090213	-0.08382	-0.16679
N	3.409384	0.636442	1.018866	-0.62432
H	4.22059	0.393497	0.451799	0.29427
H	3.490651	1.628469	1.232808	0.28447
H	3.505658	0.136045	1.901126	0.28663
P	1.69548	-2.26603	-0.26275	0.26537
H	2.658679	-3.04859	0.412039	0.02588
H	0.552303	-3.00697	0.104435	-0.02285
H	1.878912	-2.81524	-1.54812	0.01747
C	0.1369	1.621491	-0.85169	0.02507
C	-0.13971	2.365594	0.312578	0.03286
C	-0.35266	0.290383	-1.07953	-0.30484
H	-0.53919	1.876864	1.197426	0.07664
N	-1.91026	3.31421	0.019436	-0.51516
H	-2.58686	2.562033	-0.12139	0.19864
H	-2.24556	3.912945	0.775396	0.29247
H	-1.869	3.86724	-0.83657	0.27758
H	0.423213	3.276895	0.504196	0.09941
H	-0.26326	-0.04461	-2.11793	0.1374
C	-3.85936	-1.31635	0.813983	-0.11337
C	-3.80213	-1.13891	-0.56623	-0.04347
C	-2.64635	-0.63715	-1.15852	-0.21741
C	-1.52923	-0.29214	-0.38418	0.31856
C	-1.59612	-0.49473	1.002726	-0.17025
C	-2.7496	-0.99971	1.595695	-0.07585

H	-4.75928	-1.7165	1.277283	0.11762
H	-4.65751	-1.40066	-1.18676	0.10985
H	-2.60494	-0.50727	-2.2409	0.11323
H	-0.71719	-0.29438	1.619311	0.09546
H	-2.77675	-1.16042	2.67226	0.111
H	0.633365	2.135781	-1.67659	0.07445

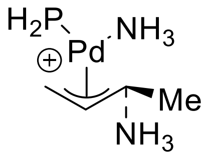

TS 10.
Gibbs Free Energy: -739.059048
Imaginary Frequency: -327.93
Cartesian coordinates and point charges:

Pd	-0.642894	0.103180	0.109908	-0.19354
N	-1.115447	2.333495	-0.006646	-0.57095
H	-0.535094	2.893555	0.616037	0.26982
H	-0.990196	2.705837	-0.947193	0.26041
H	-2.081501	2.525454	0.254439	0.28646
P	-2.795621	-0.795776	-0.313284	0.26288
H	-3.422005	-1.517270	0.723348	0.01918
H	-3.904854	0.004419	-0.670104	0.01260
H	-2.940216	-1.763690	-1.327973	0.00643
C	1.371873	-0.541110	0.683858	-0.15859
C	2.169341	0.078012	-0.320203	0.45967
C	0.501407	-1.626121	0.381288	-0.28430
H	0.139496	-2.262532	1.189273	0.15267
H	0.593254	-2.133428	-0.584881	0.12154
H	1.572341	-0.271375	1.724341	0.11363
N	3.851262	-0.994373	-0.369609	-0.62558
H	3.576443	-1.976323	-0.419275	0.28237
H	4.500458	-0.802565	-1.135091	0.31655
H	4.350278	-0.858038	0.511136	0.28750
H	1.923540	-0.181270	-1.353278	0.02459
C	2.767359	1.432603	-0.104201	-0.50568
H	3.591919	1.640686	-0.794398	0.15050
H	2.000696	2.197850	-0.280753	0.15654
H	3.122301	1.554900	0.927216	0.15530

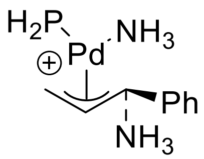

TS 11.
Gibbs Free Energy: -930.610452
Imaginary Frequency: -340.99
Cartesian coordinates and point charges:

Pd	1.438615	-0.121051	0.127361	-0.23041
N	0.451925	-2.180413	0.183590	-0.58363
H	0.968187	-2.914162	-0.298477	0.29773
H	0.339222	-2.483705	1.150101	0.27951
H	-0.482034	-2.154992	-0.226018	0.20049
P	3.698776	-0.654209	-0.359715	0.30658
H	4.359209	0.048881	-1.388703	-0.00570
H	4.658602	-0.439466	0.651003	0.00827
H	4.126841	-1.948934	-0.733377	-0.00367
C	0.119925	1.515394	0.657862	-0.03993
C	-0.903488	1.322325	-0.329801	0.16076
C	1.409013	1.984081	0.293531	-0.37729
H	2.078068	2.369981	1.062342	0.16991
H	-0.555486	1.416492	-1.362921	0.09671
H	1.569634	2.408905	-0.702094	0.14462
H	-0.149060	1.432291	1.713043	0.11030
N	-1.915334	2.960750	-0.384242	-0.68907
H	-1.279184	3.747995	-0.523742	0.35040
H	-2.636177	2.978764	-1.108672	0.31067
H	-2.373002	3.074388	0.522182	0.28544
C	-4.041652	-1.553049	0.072103	-0.08911
C	-3.557758	-1.203964	-1.186663	-0.05529
C	-2.543395	-0.256891	-1.300418	-0.15471
C	-1.996476	0.341348	-0.159281	0.18118
C	-2.49379	-0.01082	1.102074	-0.13975
C	-3.51006	-0.95305	1.214685	-0.08254
H	-4.83538	-2.29174	0.166048	0.11967
H	-3.97052	-1.66777	-2.08057	0.11453
H	-2.15672	0.011488	-2.28546	0.09342
H	-2.07954	0.440818	2.004205	0.09957
H	-3.8906	-1.22237	2.198233	0.12135

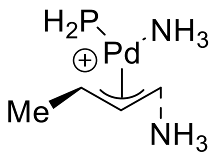

TS 12.
Gibbs Free Energy: -739.056954
Imaginary Frequency: -347.79
Cartesian coordinates and point charges:

Pd	-0.476380	-0.328403	-0.071278	-0.25590
N	-1.226266	-2.455758	-0.448558	-0.54556
H	-1.608576	-2.876857	0.396900	0.25850
H	-1.976418	-2.445644	-1.137904	0.28187
H	-0.518059	-3.095939	-0.802137	0.26301
P	-2.501095	0.753341	0.546203	0.27643
H	-3.721931	0.098904	0.825568	0.01534
H	-2.488041	1.595727	1.678186	-0.00293
H	-3.010259	1.696049	-0.372659	0.00811
C	1.591697	0.237944	-0.524620	-0.18591
C	2.233630	-0.739256	0.279930	0.07536
C	0.854849	1.315037	0.047355	0.08894
H	2.479537	-1.706770	-0.155076	0.10871
H	1.998506	-0.768970	1.344206	0.08709
H	0.989710	1.489555	1.123101	0.06330
H	1.762856	0.216586	-1.604090	0.13255
N	4.087373	-0.220263	0.545753	-0.53659
H	4.087325	0.707326	0.972601	0.28088
H	4.642823	-0.846406	1.132357	0.29722
H	4.543775	-0.143085	-0.364307	0.28192
C	0.490601	2.527904	-0.757122	-0.29581
H	-0.373454	3.051910	-0.329856	0.10808
H	1.320180	3.251066	-0.779528	0.09315
H	0.249546	2.265352	-1.795254	0.10225

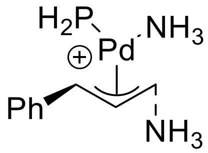

TS 13.
Gibbs Free Energy: -930.608964
Imaginary Frequency: -351.45
Cartesian coordinates and point charges:

Pd	-1.167300	-0.627673	0.061506	-0.19639

N	-2.953855	-1.723135	0.963254	-0.65986
H	-2.815537	-2.731785	0.972190	0.30029
H	-3.127276	-1.442742	1.926981	0.30663
H	-3.813334	-1.551121	0.444138	0.28274
P	0.173756	-2.393916	-0.821219	0.31347
H	-0.157216	-3.763379	-0.938129	0.00589
H	0.692314	-2.222924	-2.123254	-0.01886
H	1.407729	-2.552324	-0.152642	-0.01694
C	-0.858061	1.525496	0.393091	-0.07728
C	-2.073331	2.019892	-0.145157	0.17517
C	0.139946	0.961415	-0.457246	-0.30186
H	-2.328756	1.753925	-1.171523	0.05877
H	-0.008279	1.098467	-1.537102	0.14605
H	-0.639434	1.734092	1.442248	0.08886
N	-1.866820	3.907292	-0.514563	-0.48488
H	-1.050714	4.005264	-1.120612	0.25537
H	-2.663265	4.363918	-0.964371	0.28441
H	-1.664824	4.397686	0.357973	0.26319
H	-2.930907	2.147748	0.513470	0.06181
C	4.220396	0.148788	0.533582	-0.10283
C	3.815931	0.256194	-0.794808	-0.07869
C	2.487747	0.539687	-1.095851	-0.16558
C	1.539725	0.718959	-0.078788	0.25350
C	1.960333	0.605643	1.255371	-0.22324
C	3.287893	0.327659	1.556086	-0.02587
H	5.258542	-0.072765	0.773924	0.11352
H	4.537999	0.121857	-1.598311	0.11316
H	2.173989	0.621937	-2.138395	0.10288
H	1.241484	0.718012	2.067603	0.13055
H	3.598210	0.244350	2.596321	0.09600

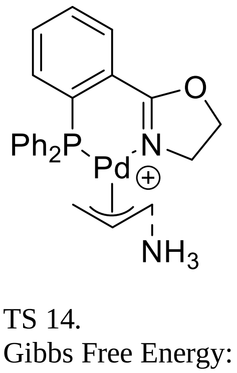

TS 14.
Gibbs Free Energy: -1581.625685
Imaginary Frequency: -350.39
Cartesian coordinates and point charges:

N	-5.780870	2.222445	0.259987	-0.53399
H	-5.399968	3.138808	0.502922	0.28064
H	-6.244466	2.309995	-0.645837	0.28524
H	-6.478813	1.960016	0.959953	0.29673
Pd	-1.582508	0.512773	-0.400494	-0.17101
N	-1.354167	-1.701765	-0.373971	-0.32196
C	-2.336771	-2.573177	0.269987	0.01982
P	0.721115	0.424106	0.056040	-0.32827
C	-3.405312	1.613075	-0.882174	-0.03803
C	-2.317622	2.454114	-0.487406	-0.33520
C	-4.308456	1.063312	0.069928	0.06138
H	-1.835206	3.084300	-1.235343	0.14014
H	-4.884192	0.179724	-0.207487	0.09068
H	-2.304342	2.879701	0.521895	0.12823
H	-4.011788	1.084296	1.120393	0.11682
C	1.487911	-1.550232	4.166543	-0.06387
C	2.298741	-1.898122	3.088470	-0.11673
C	2.090074	-1.317631	1.839813	-0.09649
C	1.060969	-0.386876	1.660511	0.26789
C	0.244168	-0.050511	2.745803	-0.16111
C	0.462202	-0.621999	3.995788	-0.09656
H	1.654831	-2.003788	5.142203	0.10724
H	3.100176	-2.623485	3.219103	0.12212
H	2.735592	-1.592613	1.004068	0.05298
H	-0.564921	0.669268	2.604189	0.12111
H	-0.171525	-0.347596	4.837751	0.10823
C	3.438737	4.141424	-0.204898	-0.09864
C	2.383227	3.957821	-1.097637	-0.07046
C	1.567020	2.837757	-0.984166	-0.19506
C	1.813516	1.881153	0.007671	0.30153
C	2.874376	2.068775	0.898295	-0.18667
C	3.680118	3.200407	0.793076	-0.05507
H	4.071979	5.023364	-0.284591	0.11275
H	2.193172	4.693455	-1.877483	0.10632
H	0.736228	2.692172	-1.677727	0.13867
H	3.073287	1.332794	1.677400	0.07650
H	4.500855	3.344717	1.493698	0.11199
C	2.666251	-2.559393	-2.941787	-0.13525
C	1.494586	-2.905540	-2.276625	-0.00606
C	0.876454	-2.005252	-1.404668	-0.21819
C	1.467186	-0.745378	-1.156197	0.33584
C	2.648571	-0.422466	-1.823088	-0.18743
C	3.239609	-1.314357	-2.717804	-0.02188

H	3.127446	-3.264952	-3.629743	0.12354
H	1.042670	-3.882086	-2.437324	0.10639
C	-0.371124	-2.436757	-0.757046	0.55367
H	3.126982	0.538960	-1.638748	0.08457
H	4.158175	-1.032781	-3.229703	0.11047
O	-0.492154	-3.757724	-0.554026	-0.42012
C	-1.784456	-3.986803	0.044179	0.29303
H	-3.323990	-2.420295	-0.185306	0.03763
H	-2.413792	-2.305471	1.334256	0.02467
H	-2.375784	-4.584803	-0.657855	0.03562
H	-1.626106	-4.559161	0.962592	0.01318
H	-3.645206	1.493296	-1.940496	0.09245

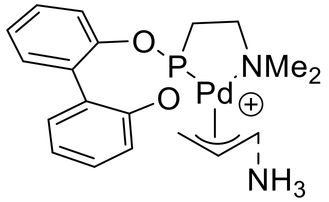

TS 15.
Gibbs Free Energy: -1466.472003
Imaginary Frequency: -355.67
Cartesian coordinates and point charges:

Pd	1.864663	-0.216322	-0.396740	-0.29811
N	2.130784	-2.157094	0.866745	0.07790
C	0.886623	-2.462480	1.614093	0.06103
P	-0.359845	-0.677683	0.001681	0.58694
C	3.445273	0.981178	-1.343211	0.01542
C	4.224582	1.298092	-0.201501	0.02614
C	2.131651	1.512669	-1.525075	-0.32428
H	1.661559	1.456403	-2.506691	0.15474
H	1.801236	2.362499	-0.918538	0.10671
H	3.918392	0.411901	-2.145610	0.09126
H	3.728020	1.777867	0.643649	0.12256
O	-1.080867	0.127428	1.243337	-0.44672
O	-1.511447	-0.673339	-1.148125	-0.46022
C	-5.578642	-1.076288	-0.394790	-0.10377
C	-4.879782	-2.093089	-1.041451	-0.08153
C	-3.511911	-1.960994	-1.260918	-0.21615
C	-2.861050	-0.814291	-0.828587	0.37400
C	-3.534116	0.224814	-0.173012	-0.03786
C	-4.910139	0.065526	0.031947	-0.11367
H	-6.647531	-1.175253	-0.216712	0.11922
H	-5.398205	-2.988254	-1.379404	0.11709

H	-2.942307	-2.726063	-1.786722	0.14793
H	-5.453995	0.851013	0.556485	0.11295
C	-2.743119	3.862118	0.529496	-0.10345
C	-1.523435	3.761689	1.196120	-0.09553
C	-0.953581	2.510107	1.411186	-0.18569
C	-1.615521	1.376914	0.959524	0.31310
C	-2.838008	1.445796	0.279472	0.02976
C	-3.389088	2.716283	0.077577	-0.13006
H	-3.192541	4.838409	0.358287	0.11888
H	-1.019581	4.657402	1.555050	0.11531
H	-0.012084	2.389946	1.946292	0.13146
H	-4.333882	2.799055	-0.459444	0.11452
C	-0.370309	-2.345942	0.762712	0.04676
C	2.449831	-3.236158	-0.077007	-0.25937
H	3.391194	-3.003976	-0.587862	0.11434
H	2.556837	-4.203770	0.446513	0.08997
H	1.669209	-3.329457	-0.839470	0.12375
C	3.231175	-2.011619	1.825302	-0.28202
H	4.170450	-1.850874	1.283524	0.11534
H	3.042179	-1.148735	2.475464	0.09080
H	3.339571	-2.915152	2.452319	0.10909
H	5.048468	0.641553	0.076489	0.09036
H	0.824799	-1.745463	2.445924	0.05460
H	0.967370	-3.471167	2.060387	0.01927
H	-1.271302	-2.483737	1.375904	-0.00265
H	-0.398867	-3.093620	-0.043247	0.02385
N	5.346481	2.814990	-0.611654	-0.50460
H	4.727163	3.564013	-0.924879	0.27538
H	5.926811	3.168432	0.152062	0.28700
H	5.955258	2.569148	-1.393521	0.26825

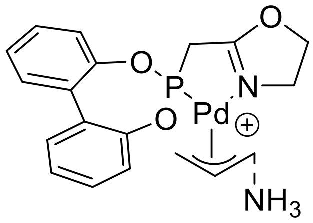

TS 16.
Gibbs Free Energy: -1539.310705
Imaginary Frequency: -353.53
Cartesian coordinates and point charges:

Pd	-1.866139	-0.435830	-0.101173	-0.27878
N	-2.285570	1.742077	-0.448881	-0.31287

C	-1.325196	2.526210	-0.792456	0.57593
P	0.288221	0.383583	-0.364020	0.60771
C	-3.271234	-2.101083	0.127623	0.05171
C	-4.021337	-1.581943	1.215327	0.04630
C	-1.907233	-2.488485	0.277777	-0.37570
H	-1.491942	-2.640888	1.278708	0.14940
H	-3.791633	-2.287925	-0.813448	0.08932
H	-3.484842	-1.297853	2.121455	0.12911
O	1.150461	0.554963	1.011579	-0.47511
O	1.419776	-0.108456	-1.441349	-0.42260
C	3.664167	-3.476031	-0.523727	-0.12204
C	2.621203	-3.566908	-1.443719	-0.07455
C	1.852862	-2.442971	-1.732267	-0.21053
C	2.144891	-1.245220	-1.096326	0.31692
C	3.179907	-1.121843	-0.162426	0.02005
C	3.937358	-2.267370	0.107885	-0.11041
H	4.265857	-4.352912	-0.292722	0.12403
H	2.408771	-4.511421	-1.941133	0.11362
H	1.040353	-2.470532	-2.457290	0.15727
H	4.741851	-2.204069	0.840360	0.10928
C	5.098992	1.836295	1.212577	-0.09861
C	4.075541	2.628444	1.727389	-0.10669
C	2.754804	2.202011	1.627370	-0.18294
C	2.473195	0.990857	1.009966	0.37105
C	3.480333	0.171817	0.481890	-0.04819
C	4.799626	0.624415	0.599852	-0.10266
H	6.133708	2.165518	1.282334	0.11910
H	4.302606	3.576376	2.211342	0.12223
H	1.934993	2.783286	2.047559	0.14289
H	5.599627	0.015221	0.179576	0.11025
C	0.079734	2.100537	-1.023727	-0.18564
H	-4.906184	-0.981476	1.007105	0.09076
H	0.789410	2.809674	-0.574034	0.06957
H	0.290478	2.085458	-2.103123	0.11523
O	-1.611690	3.813330	-0.973037	-0.41318
C	-3.029533	3.972904	-0.718701	0.23491
C	-3.503366	2.553368	-0.362827	0.03049
H	-1.429969	-3.086230	-0.498883	0.14049
H	-3.138116	4.698896	0.092601	0.04562
H	-3.480925	4.378951	-1.628842	0.03997
H	-4.252641	2.165702	-1.064262	0.05183
H	-3.923839	2.482985	0.648308	0.03935
N	-4.990323	-3.031962	2.055024	-0.56426

H	-4.300939	-3.740255	2.311248	0.28464
H	-5.529743	-2.785893	2.887561	0.30344
H	-5.623209	-3.447682	1.370441	0.28231

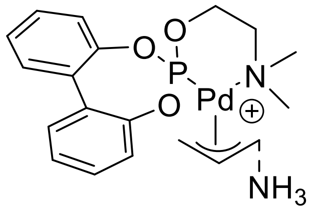

TS 17.
Gibbs Free Energy: -1541.679241
Imaginary Frequency: -352.77
Cartesian coordinates and point charges:

C	-1.69094	2.021803	1.010183	-0.23661
H	-1.42296	1.862706	2.059347	0.13818
C	-3.05176	1.883673	0.597165	-0.0077
C	-4.01086	1.232504	1.41401	0.01502
H	-3.65258	0.648838	2.263372	0.12499
H	-3.40028	2.380595	-0.31037	0.10023
Pd	-1.78988	0.156822	0.089912	-0.37161
N	-2.52232	-1.75499	-1.00553	0.18891
P	0.338871	-0.69767	0.023091	0.80798
H	-4.19009	3.054819	3.019876	0.27581
N	-4.9143	2.551179	2.505409	-0.53231
H	-5.59691	2.188843	3.174438	0.29733
C	-0.31586	-3.04855	-1.08658	0.18612
C	-1.44459	-2.34648	-1.82291	-0.05244
H	-0.67443	-3.95745	-0.58832	0.04077
H	0.418344	-3.35464	-1.84434	0.05997
C	-3.58601	-1.31314	-1.9165	-0.26219
H	-3.18584	-0.57607	-2.62309	0.11045
C	-3.06303	-2.72136	-0.04498	-0.17048
H	-3.40082	-3.64536	-0.54965	0.07265
H	-2.30917	-2.97586	0.708272	0.09625
H	-3.92094	-2.27217	0.469551	0.0571
H	-4.39257	-0.84384	-1.34126	0.09928
H	-1.04771	-1.54712	-2.46653	0.06586
O	0.378973	-2.30928	-0.08287	-0.4005
O	1.326596	-0.43179	1.267929	-0.47646
O	1.280458	-0.31828	-1.26745	-0.42667
C	1.883567	3.180407	-2.16613	-0.08329
C	1.296949	1.919791	-2.11173	-0.16066

C	1.834981	0.956716	-1.26907	0.28299
C	2.946802	1.209706	-0.4559	0.00876
H	1.475882	3.940702	-2.82983	0.11015
C	4.598548	-1.86808	2.038406	-0.06567
C	3.221719	-1.67358	2.000909	-0.22621
C	2.702319	-0.66909	1.199339	0.38592
C	3.5112	0.156925	0.410556	-0.0392
H	5.01677	-2.6541	2.664155	0.11402
C	2.995863	3.460708	-1.37409	-0.10877
C	3.518512	2.485296	-0.53158	-0.1104
C	5.434639	-1.05613	1.274651	-0.12833
C	4.893449	-0.0597	0.470821	-0.09295
H	-5.38378	3.220979	1.894542	0.27638
H	-1.03762	2.719552	0.4846	0.07458
H	-4.92278	0.847923	0.957293	0.10401
H	-1.87416	-3.11567	-2.49524	0.03075
H	-4.00407	-2.16287	-2.48675	0.08986
H	0.437899	1.658926	-2.72925	0.12692
H	2.539231	-2.28109	2.592202	0.15737
H	3.458062	4.445305	-1.41135	0.11863
H	4.377827	2.713521	0.098762	0.10433
H	6.512102	-1.20699	1.29622	0.12328
H	5.54688	0.554648	-0.14846	0.10757

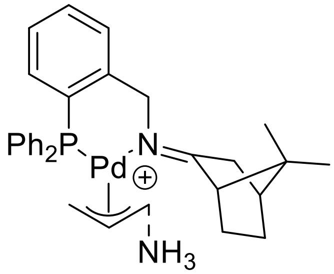

TS 18.
Gibbs Free Energy: -1780.032119
Imaginary Frequency: -347.81
Cartesian coordinates and point charges:

C	0.899509	3.294089	0.823943	-0.37371
H	1.647401	3.696783	0.131769	0.13947
C	-0.47635	3.64581	0.656718	-0.06575
C	-0.96262	4.245011	-0.5422	0.08414
H	-0.31801	4.205921	-1.42226	0.10371
H	-1.16126	3.581822	1.505415	0.10515
Pd	-0.00661	1.651592	-0.07974	-0.06727

N	-1.31536	0.242041	-1.17022	-0.49693
P	1.467687	-0.19225	-0.02096	-0.14162
H	-1.54372	6.359224	0.426677	0.26258
N	-0.9405	6.100795	-0.35585	-0.44131
H	0.016309	6.360748	-0.10916	0.25405
C	0.198732	-2.74061	3.632784	-0.13928
C	0.559494	-1.39948	3.772146	-0.017
C	0.936073	-0.66178	2.656379	-0.23271
C	0.979646	-1.25819	1.38799	0.42879
C	0.61005	-2.59962	1.255498	-0.21939
C	0.220809	-3.33538	2.37513	-0.01929
H	-0.10075	-3.31872	4.505398	0.11406
H	0.540845	-0.92746	4.753203	0.09737
H	1.205933	0.390795	2.76675	0.10236
H	0.619075	-3.07951	0.276293	0.07698
H	-0.06638	-4.37951	2.2586	0.09586
C	6.001026	0.56446	0.442159	-0.07407
C	5.236742	1.290968	-0.47036	-0.09707
C	3.870895	1.052268	-0.57589	-0.13331
C	3.259868	0.072894	0.215453	0.08989
C	4.031943	-0.65349	1.127106	0.00367
C	5.397771	-0.40332	1.241031	-0.12205
H	7.06901	0.75593	0.531979	0.10661
H	5.706448	2.048837	-1.09534	0.11232
H	3.266707	1.6287	-1.28044	0.10056
H	3.568049	-1.41789	1.751888	-0.01055
H	5.992452	-0.97155	1.954507	0.12427
C	1.189343	-3.2797	-3.46638	-0.06306
C	0.222339	-2.2862	-3.35699	-0.14861
C	0.295768	-1.29376	-2.37393	-0.00656
C	1.36534	-1.33415	-1.45744	0.14949
C	2.343267	-2.32771	-1.5891	-0.13804
C	2.266261	-3.29337	-2.58585	-0.07653
H	1.102746	-4.03679	-4.24383	0.1111
H	-0.61092	-2.2704	-4.06143	0.09771
H	3.182083	-2.34857	-0.892	0.07734
H	3.039608	-4.05482	-2.66854	0.11015
C	-0.72946	-0.18207	-2.44078	0.20387
H	-1.52257	-0.48283	-3.1432	0.02717
C	-2.32818	-0.38927	-0.70998	0.39062
C	-3.02935	-1.60142	-1.29053	-0.03074
C	-4.15768	-1.81496	-0.26739	-0.02441
C	-2.72745	-1.13956	1.561974	-0.02727

C	-3.49553	-2.36733	1.007349	-0.10484
H	-3.40096	-1.40467	-2.30753	0.04349
H	-2.3421	-2.45864	-1.36405	0.00225
H	-4.99496	-2.41593	-0.64578	0.00986
H	-1.64659	-1.30997	1.62858	-0.02206
H	-3.06493	-0.85361	2.566204	0.02052
H	-2.82558	-3.20826	0.779918	0.04294
H	-4.23877	-2.74204	1.721663	0.03472
C	-3.06071	-0.02517	0.545228	-0.08829
C	-4.51966	-0.35948	0.133083	0.45816
C	-5.03195	0.503348	-1.01926	-0.50704
H	-5.98608	0.112887	-1.40155	0.12592
H	-5.21635	1.529341	-0.66897	0.12143
H	-4.33951	0.574866	-1.86847	0.08936
C	-5.53195	-0.25605	1.266994	-0.36273
H	-6.51511	-0.61024	0.925039	0.07877
H	-5.26926	-0.83417	2.158763	0.10579
H	-5.6536	0.791555	1.577859	0.08056
H	-1.2259	6.635889	-1.17965	0.27817
H	-2.02391	4.147704	-0.77384	0.0547
H	1.286683	3.10847	1.827036	0.13325
H	-0.25049	0.708484	-2.87523	0.00795
H	-2.84987	0.997505	0.88933	-0.00567

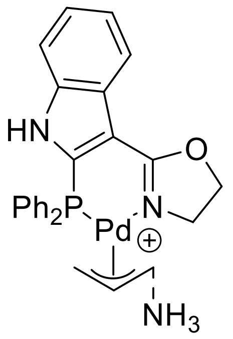

TS 19.
Gibbs Free Energy: -1713.095535
Imaginary Frequency: -351.35
Cartesian coordinates and point charges:

C	3.634355	0.70085	-0.44403	-0.38009
H	3.557686	1.024333	-1.4879	0.14665
C	4.146802	-0.60018	-0.14489	0.0057
C	4.259636	-1.60535	-1.14529	0.01845
H	4.586468	-0.80069	0.834148	0.09076
Pd	1.968999	-0.46158	0.018176	-0.1507

N	0.661237	-2.19084	0.496778	-0.4535
P	0.091821	0.926852	0.121182	-0.13966
H	6.173567	-2.20179	-2.65787	0.29306
N	5.992096	-1.54246	-1.89764	-0.50359
H	6.677113	-1.70182	-1.15742	0.26788
C	1.181162	-3.39676	1.142146	0.09216
C	-0.49917	4.621735	-2.58505	-0.1
C	0.163998	3.487962	-3.05406	-0.07048
C	0.36025	2.399916	-2.21033	-0.18041
C	-0.12133	2.429983	-0.89604	0.2185
C	-0.78512	3.570034	-0.42993	-0.15537
C	-0.96894	4.663357	-1.27442	-0.05575
H	-0.64614	5.476429	-3.24299	0.11418
H	0.532715	3.455594	-4.07795	0.11034
H	0.883643	1.51163	-2.5712	0.11251
H	-1.15633	3.606486	0.595328	0.06444
H	-1.48169	5.550122	-0.90542	0.11047
C	-0.8406	2.330288	4.41982	-0.10513
C	-1.82124	1.641746	3.710931	-0.04529
C	-1.57009	1.204353	2.411832	-0.17683
C	-0.32873	1.452149	1.81928	0.27603
C	0.659201	2.131555	2.542489	-0.19682
C	0.400754	2.576488	3.833928	-0.06088
H	-1.04098	2.672439	5.433686	0.11384
H	-2.78973	1.445003	4.167817	0.10236
H	-2.34685	0.666217	1.866767	0.08239
H	1.635354	2.315099	2.088053	0.12946
H	1.171431	3.10983	4.38795	0.10688
C	-0.61277	-2.3505	0.343809	0.6571
O	-1.11239	-3.542	0.715088	-0.35168
C	-0.00264	-4.36884	1.114642	0.18012
H	2.052365	-3.77505	0.59196	0.0131
H	-0.24775	-4.81383	2.08298	0.03784
H	0.104285	-5.16428	0.367438	0.04374
C	-1.57235	-1.40928	-0.18609	-0.39375
C	-1.35392	-0.05137	-0.37738	0.33191
N	-2.48632	0.502269	-0.90597	-0.54878
C	-3.45654	-0.46638	-1.07252	0.3096
C	-2.92091	-1.691	-0.61212	0.1317
C	-3.72178	-2.84381	-0.67037	-0.19119
C	-5.00281	-2.73433	-1.18198	-0.08519
C	-5.51053	-1.50371	-1.64146	-0.09095
C	-4.74525	-0.35152	-1.59599	-0.25445

H	-3.34318	-3.79856	-0.31444	0.12239
H	-5.63603	-3.61847	-1.22862	0.1096
H	-6.5234	-1.45778	-2.03719	0.11618
H	-5.13165	0.603136	-1.94984	0.15169
H	1.513658	-3.15233	2.161115	0.05219
H	-2.57406	1.479575	-1.16347	0.3361
H	6.128568	-0.59252	-2.24735	0.27502
H	3.727125	-1.44834	-2.0848	0.11061
H	4.306128	-2.64927	-0.83503	0.11047
H	3.817662	1.51756	0.255887	0.14509

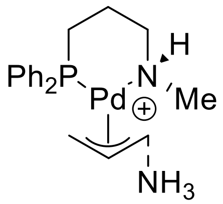

TS 20.
Gibbs Free Energy: -1317.236330
Imaginary Frequency: -348.64
Cartesian coordinates and point charges:

C	1.981679	0.951793	-1.64706	-0.20059
H	2.038905	1.918194	-1.13307	0.08516
C	3.081613	0.039796	-1.56676	-0.14184
C	4.122863	0.205962	-0.61102	0.11183
H	3.216897	-0.71828	-2.341	0.10887
Pd	1.39475	-0.48355	-0.26661	-0.2502
N	1.362588	-2.29709	1.1452	-0.11261
P	-0.80184	0.07084	0.366234	-0.06077
H	5.865346	0.705215	-2.19438	0.2685
N	5.513048	1.208663	-1.37849	-0.4635
H	5.098972	2.082335	-1.70902	0.26431
C	-4.26581	-1.89934	-1.96293	-0.0643
C	-4.50105	-1.40368	-0.68042	-0.10353
C	-3.47027	-0.79944	0.031854	-0.11545
C	-2.19288	-0.69001	-0.53146	0.24871
C	-1.96618	-1.19053	-1.81726	-0.17954
C	-3.00013	-1.78962	-2.53281	-0.09633
H	-5.07373	-2.37371	-2.5174	0.11207
H	-5.49108	-1.48774	-0.23517	0.11467
H	-3.66739	-0.40125	1.028898	0.10272
H	-0.96834	-1.10961	-2.25392	0.14195

H	-2.81659	-2.17575	-3.53405	0.11145
C	-1.66621	4.601997	0.680133	-0.1057
C	-0.57131	4.032021	1.32995	-0.08289
C	-0.33359	2.666804	1.221842	-0.14773
C	-1.19979	1.849712	0.483177	0.15709
C	-2.29069	2.428907	-0.17064	-0.14766
C	-2.51853	3.800819	-0.07289	-0.05077
H	-1.85001	5.672077	0.758946	0.11408
H	0.09996	4.655497	1.918726	0.10559
H	0.541606	2.23177	1.711139	0.11755
H	-2.96702	1.812526	-0.76305	0.05855
H	-3.37029	4.242847	-0.5873	0.11028
C	-0.87816	-2.01989	2.319044	0.14335
C	-0.98887	-0.50384	2.113725	-0.13952
C	0.003973	-2.83178	1.372773	0.04656
H	1.847343	-2.97992	0.563583	0.23424
H	-1.88	-2.46474	2.233019	-0.01559
H	-0.57014	-2.20235	3.359524	0.00073
H	-0.19273	0.023233	2.660863	0.07048
H	-0.47192	-2.91465	0.385424	-0.02291
C	2.130756	-2.13531	2.382931	-0.19838
H	1.726346	-1.30029	2.966501	0.10649
H	3.171656	-1.89774	2.135113	0.06497
H	2.110171	-3.04171	3.010971	0.08549
H	0.069556	-3.85435	1.785442	0.02606
H	-1.93378	-0.13582	2.539639	0.04747
H	1.370612	0.971736	-2.55084	0.10072
H	4.729834	-0.66025	-0.34483	0.06831
H	3.939095	0.886686	0.222652	0.09318
H	6.301922	1.429573	-0.76608	0.27841

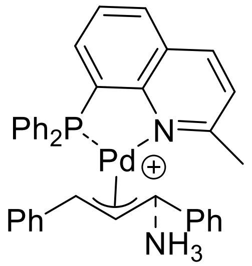

TS 21.
Gibbs Free Energy: -2006.153699
Imaginary Frequency: -265.98

Cartesian coordinates and point charges:

P	1.466722	0.743864	0.199137	0.02256
N	-1.17149	1.764606	-0.61844	-0.44063
C	-2.32579	2.071195	-1.19066	0.49435
Pd	-0.55543	-0.39794	-0.05281	-0.21908
C	-0.35797	-2.36699	0.599545	-0.26637
C	-1.71366	-2.25743	0.151568	-0.0374
C	-2.74975	-1.90766	1.090409	0.0977
N	-3.29779	-3.41892	1.904102	-0.62184
H	-2.02137	-2.64869	-0.82143	0.06505
H	-2.48135	-3.87965	2.313421	0.33305
H	-3.69401	-4.04349	1.198415	0.28824
H	-4.00289	-3.26392	2.629908	0.3085
H	-0.19412	-2.28929	1.684268	0.10586
H	-2.37316	-1.4124	1.9925	0.12979
C	0.738092	-3.05107	-0.10631	0.21209
C	0.618701	-3.54359	-1.41359	-0.25679
C	1.980924	-3.16421	0.536773	-0.09974
C	1.708326	-4.1236	-2.05571	-0.00927
H	-0.33111	-3.46446	-1.94344	0.12908
C	3.071811	-3.7332	-0.10927	-0.08586
H	2.095311	-2.77192	1.550408	0.03927
C	2.941024	-4.21701	-1.41027	-0.11566
H	1.594213	-4.50451	-3.06987	0.08815
H	4.029489	-3.79576	0.406322	0.09774
H	3.792606	-4.66722	-1.91746	0.10357
C	-4.02753	-1.32072	0.604741	0.1713
C	-4.50977	-0.1495	1.194653	-0.1904
C	-4.75107	-1.90971	-0.43805	-0.18036
C	-5.69295	0.43065	0.745069	-0.04545
H	-3.93926	0.323064	1.99614	0.08679
C	-5.93886	-1.33755	-0.87979	-0.07018
H	-4.37908	-2.81596	-0.92099	0.12366
C	-6.41133	-0.16524	-0.28926	-0.07987
H	-6.05626	1.347695	1.205982	0.10195
H	-6.49623	-1.80447	-1.68986	0.10952
H	-7.34174	0.280928	-0.63604	0.11014
C	4.250926	-0.51592	3.652053	-0.12718
C	4.742691	-0.50018	2.35066	-0.01701
C	3.93669	-0.06316	1.299833	-0.16988
C	2.627503	0.357917	1.548769	0.22739
C	2.134338	0.328555	2.860896	-0.12884
C	2.944462	-0.09447	3.907429	-0.06288

H	4.883432	-0.85453	4.470967	0.10869
H	5.761529	-0.82644	2.14709	0.09686
H	4.329049	-0.06082	0.28217	0.06217
H	1.105576	0.639108	3.059318	0.06024
H	2.557596	-0.10138	4.925438	0.10056
C	4.136585	0.792013	-3.55756	-0.09256
C	4.067411	1.940497	-2.7699	-0.0408
C	3.253884	1.963613	-1.64074	-0.21119
C	2.49868	0.83758	-1.29989	0.24619
C	2.561455	-0.30789	-2.10084	-0.21848
C	3.385561	-0.33243	-3.22206	-0.03506
H	4.776384	0.776724	-4.43855	0.10558
H	4.651842	2.820244	-3.03486	0.09932
H	3.210408	2.862695	-1.02461	0.09622
H	1.965243	-1.18516	-1.83937	0.14821
H	3.434479	-1.23091	-3.83604	0.07867
C	-0.42592	2.750454	-0.01798	0.33738
C	0.867573	2.443425	0.500478	-0.13572
C	1.586648	3.421732	1.158085	0.07762
C	1.078021	4.72861	1.308948	-0.14703
C	-0.14369	5.054237	0.771715	-0.15903
C	-0.91351	4.080014	0.093743	0.00676
H	2.569223	3.179724	1.56704	0.01408
H	1.664845	5.478055	1.836317	0.14172
H	-0.53966	6.066106	0.859875	0.13158
C	-2.86062	3.384685	-1.13835	-0.31655
C	-2.17281	4.369341	-0.48502	-0.01943
H	-3.821	3.582967	-1.6122	0.1445
H	-2.57391	5.380473	-0.41033	0.12519
C	-3.06548	1.007123	-1.931	-0.26099
H	-2.63011	0.020744	-1.7268	0.08756
H	-4.13096	0.995435	-1.66324	0.06414
H	-3.00473	1.193621	-3.01277	0.08251

Supplementary Table 2. Coordinates for the DFT optimized structures used to fit the oxazole moiety

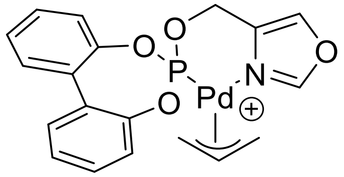

TS 22.
Gibbs Free Energy: -1556.856130
Cartesian coordinates and point charges:

C	0.492092	2.305309	-1.47114	-0.13527
H	0.503473	1.897082	-2.48541	0.1536
C	1.630044	2.981227	-0.96478	0.10281
C	2.900079	2.531388	-1.331	-0.29647
H	3.061516	2.080794	-2.31345	0.18737
H	1.51769	3.636135	-0.0984	0.12451
Pd	1.692445	0.8335	-0.4749	-0.24239
P	-0.02335	-0.59673	0.093103	0.91585
C	1.54585	-2.06474	1.561332	0.06238
H	1.539865	-3.06781	1.997982	0.11796
H	1.326832	-1.35072	2.370307	0.07636
O	0.482574	-2.04505	0.599263	-0.36249
O	-1.07816	-0.93631	-1.05632	-0.46436
O	-0.93817	-0.15602	1.362951	-0.44058
C	-2.32292	3.233183	1.5169	-0.06921
C	-1.46672	2.152802	1.70773	-0.15913
C	-1.79065	0.927606	1.142228	0.31322
C	-2.952	0.726011	0.387112	0.00119
H	-2.08741	4.198029	1.961919	0.10963
C	-3.97876	-3.16173	-1.12101	-0.05328
C	-2.67957	-2.68697	-1.27087	-0.2121
C	-2.36739	-1.42696	-0.78903	0.3642
C	-3.30004	-0.60592	-0.14723	-0.06835
H	-4.23907	-4.14975	-1.49488	0.11704
C	-3.48238	3.070947	0.759042	-0.09409
C	-3.79241	1.832216	0.207567	-0.09965
C	-4.93897	-2.36937	-0.49563	-0.12441
C	-4.59969	-1.11034	-0.01407	-0.06901
H	-0.49606	2.532114	-1.07005	0.031
H	3.784032	2.904388	-0.81668	0.1609
H	-0.55945	2.236798	2.305408	0.11747
H	-1.90776	-3.271	-1.76819	0.15354
H	-4.15198	3.91387	0.600496	0.11808
H	-4.69624	1.712893	-0.38931	0.11001

H	-5.95515	-2.73802	-0.37219	0.1261
H	-5.34567	-0.50602	0.501496	0.10449
C	2.875027	-1.78338	0.948805	0.05657
C	4.016652	-2.50728	1.001656	0.00448
C	4.408497	-0.70745	-0.09911	0.28624
N	3.153213	-0.61893	0.229805	-0.14322
O	4.989577	-1.81798	0.340741	-0.19004
H	4.298159	-3.45638	1.436404	0.17871
H	5.005606	-0.00634	-0.66923	0.13035

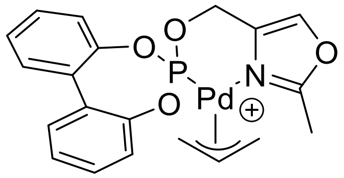

TS 23.
Gibbs Free Energy: -1596.128395
Cartesian coordinates and point charges:

C	0.312283	2.102724	-1.67415	-0.10558
H	0.207439	1.580525	-2.6291	0.14536
C	1.514451	2.78944	-1.36716	0.10228
C	2.728719	2.250911	-1.78896	-0.30185
H	2.783974	1.655855	-2.70348	0.1831
H	1.505358	3.553105	-0.5869	0.1177
Pd	1.544893	0.705462	-0.62268	-0.23079
P	-0.20333	-0.5705	0.165531	0.90266
C	1.332563	-2.6608	0.098081	0.07022
H	1.181196	-2.63876	-0.99324	0.07911
H	1.291749	-3.70631	0.417092	0.10355
O	0.236246	-2.0073	0.755443	-0.34984
O	-1.30711	-0.83631	-0.98081	-0.47517
O	-1.06267	-0.0835	1.437996	-0.46682
C	-2.35785	3.340246	1.562294	-0.046
C	-1.51894	2.243574	1.737091	-0.20393
C	-1.89951	1.015072	1.216683	0.39592
C	-3.0982	0.826253	0.520287	-0.05468
H	-2.07761	4.308644	1.972094	0.10874
C	-4.23207	-3.03471	-0.9726	-0.06167
C	-2.93496	-2.57063	-1.16799	-0.22836
C	-2.5887	-1.32028	-0.68312	0.36092
C	-3.48874	-0.50037	0.004818	-0.0294
H	-4.51802	-4.01484	-1.34863	0.11775

C	-3.55815	3.189234	0.869	-0.11787
C	-3.92186	1.94744	0.358813	-0.06782
C	-5.15815	-2.24146	-0.29857	-0.10979
C	-4.78734	-0.99145	0.183884	-0.09311
H	-0.62427	2.419508	-1.21323	0.01473
H	3.668869	2.660232	-1.42491	0.16203
H	-0.58203	2.316643	2.288153	0.12508
H	-2.19195	-3.15653	-1.70662	0.15863
H	-4.2159	4.044146	0.725749	0.12108
H	-4.8549	1.837192	-0.19323	0.10093
H	-6.17218	-2.6021	-0.13919	0.12351
H	-5.50625	-0.38456	0.73383	0.1128
C	2.642158	-2.06023	0.468368	0.07241
C	3.710172	-2.627	1.06898	-0.06484
C	4.176011	-0.55258	0.666527	0.54267
N	2.961773	-0.72409	0.21408	-0.23144
O	4.679953	-1.67628	1.188396	-0.21027
H	3.936138	-3.61246	1.452078	0.1981
C	5.017464	0.659288	0.666154	-0.4234
H	5.788697	0.581684	1.438263	0.14843
H	5.518949	0.784955	-0.30261	0.14899
H	4.405725	1.547068	0.856858	0.15591

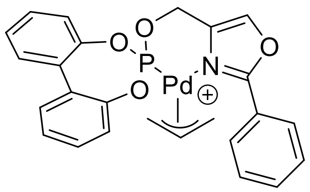

TS 24.
Gibbs Free Energy: -1787.671865
Cartesian coordinates and point charges:

C	-0.241	1.984342	-1.54745	-0.12169
H	-0.37929	1.588201	-2.55733	0.14248
C	1.014465	2.513941	-1.15787	0.08769
C	2.175528	1.927751	-1.65276	-0.27319
H	2.181373	1.455245	-2.63818	0.19916
H	1.068546	3.168092	-0.28542	0.11954
Pd	0.844248	0.35651	-0.67421	-0.25645
P	-1.06416	-0.67584	0.110182	1.01368
C	0.168063	-2.96219	0.112434	0.11177
H	0.055034	-2.974	-0.98292	0.06402
H	-0.00499	-3.9786	0.477682	0.09486

O	-0.8697	-2.16261	0.701285	-0.38347
O	-2.22332	-0.74247	-1.01011	-0.51375
O	-1.80905	-0.05703	1.402065	-0.50966
C	-2.55581	3.519422	1.620608	-0.07345
C	-1.89711	2.301545	1.760354	-0.17629
C	-2.46922	1.158675	1.219401	0.40376
C	-3.69459	1.174542	0.54375	-0.04488
H	-2.12267	4.422911	2.045486	0.11404
C	-5.46744	-2.43283	-0.96112	-0.03212
C	-4.11591	-2.18423	-1.17839	-0.25936
C	-3.56037	-1.01282	-0.69015	0.3991
C	-4.30242	-0.06481	0.021434	-0.06039
H	-5.9162	-3.34894	-1.33956	0.10982
C	-3.77278	3.573369	0.941892	-0.11615
C	-4.33382	2.414359	0.416078	-0.07449
C	-6.23918	-1.5072	-0.26203	-0.13845
C	-5.66034	-0.34002	0.222649	-0.05828
H	-1.15069	2.3299	-1.05491	0.0249
H	3.145498	2.177268	-1.22728	0.1227
H	-0.95337	2.215145	2.297986	0.11733
H	-3.48884	-2.87713	-1.73677	0.16583
H	-4.28961	4.523676	0.824119	0.12329
H	-5.28065	2.462826	-0.12115	0.10152
H	-7.29529	-1.6996	-0.08492	0.1267
H	-6.26031	0.369364	0.792229	0.10187
C	1.530327	-2.50777	0.496211	-0.01289
C	2.43269	-3.12488	1.289177	0.02605
C	3.320696	-1.30169	0.534253	0.41299
N	2.108471	-1.3221	0.031951	-0.15984
O	3.561844	-2.36766	1.314769	-0.23412
H	2.43914	-4.04192	1.862322	0.17656
C	6.569889	1.332761	-0.13597	-0.06342
C	6.278093	0.910131	1.160151	-0.07145
C	5.209414	0.05163	1.38894	-0.11843
C	4.414941	-0.36574	0.315407	-0.01771
C	4.716277	0.047874	-0.98666	-0.03461
C	5.795025	0.895167	-1.20945	-0.05722
H	7.414655	1.996158	-0.31231	0.11003
H	6.890702	1.24491	1.994745	0.11542
H	4.982609	-0.29197	2.397284	0.12417
H	4.119079	-0.32264	-1.81971	0.05841
H	6.042209	1.203967	-2.22374	0.09402

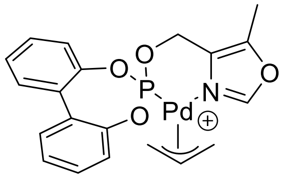

TS 25.
Gibbs Free Energy: -1596.125961
Cartesian coordinates and point charges:

C	0.055207	-2.55369	-1.35642	-0.13614
H	0.028687	-2.21539	-2.39577	0.14922
C	-0.99976	-3.34637	-0.84056	0.11982
C	-2.30425	-3.09998	-1.27403	-0.32184
H	-2.48705	-2.74005	-2.28958	0.19081
H	-0.83403	-3.92347	0.071426	0.11808
Pd	-1.37502	-1.20262	-0.49873	-0.23234
P	0.103225	0.485454	0.024768	0.96447
C	-1.71323	1.791546	1.349146	0.08364
H	-1.8546	2.805314	1.735381	0.12445
H	-1.43085	1.153439	2.200869	0.07107
O	-0.6115	1.874583	0.42941	-0.39128
O	1.139852	0.90153	-1.11743	-0.4783
O	1.03068	0.248235	1.340184	-0.4456
C	2.859118	-2.9036	1.725662	-0.06868
C	1.857294	-1.94328	1.831091	-0.14032
C	2.029731	-0.71682	1.204914	0.28874
C	3.17707	-0.39814	0.468941	0.00371
H	2.742467	-3.86679	2.218738	0.10718
C	3.710061	3.498013	-1.25666	-0.08227
C	2.493577	2.839668	-1.40639	-0.18839
C	2.340175	1.58013	-0.85173	0.34879
C	3.355623	0.936487	-0.1371	-0.04769
H	3.844891	4.48794	-1.68754	0.12324
C	4.010693	-2.62325	0.990722	-0.09824
C	4.166589	-1.38524	0.376219	-0.09352
C	4.748648	2.885833	-0.55843	-0.10812
C	4.569013	1.623207	-0.00554	-0.09081
H	1.048441	-2.61707	-0.9107	0.02609
H	-3.14855	-3.55811	-0.76193	0.16736
H	0.948999	-2.12014	2.406534	0.11202
H	1.66603	3.279845	-1.95916	0.15138
H	4.794334	-3.37261	0.898861	0.11862
H	5.064909	-1.17461	-0.2035	0.106
H	5.700371	3.398349	-0.43462	0.12539

H	5.373591	1.159811	0.564938	0.10914
C	-2.96329	1.305956	0.703542	-0.04717
C	-4.19103	1.884869	0.659868	0.31951
C	-4.27976	-0.03408	-0.33388	0.26337
N	-3.04576	0.068896	0.056724	-0.16001
O	-5.02383	1.015529	0.000078	-0.24233
H	-4.74737	-0.84397	-0.88029	0.14006
C	-4.78068	3.147971	1.140904	-0.41497
H	-5.26164	3.69056	0.317963	0.16958
H	-5.54187	2.963737	1.909432	0.15318
H	-4.01045	3.795039	1.572346	0.13312

Supplementary Table 3. Added TSFF parameters to the standard MM3* to described the Pd-catatlyzed allylic amination reaction

```
C  PdTS_Core OPT
 9  Pd(-C0-C0(-1)-C0(.1)[.NX])
-2
 1   1   2              2.1050     1.1549    -1.8195
 1   1   3              2.1805     1.7263    -2.5539
 1   1   4              2.7857     0.9661    -3.2640
 1   2   3              1.4082     5.5257    -1.2122
 1   2  C2               1.4739     3.8668    -1.4341
 1   2  C3               1.4958     4.5477     0.7841
 1   2  H1               1.0960     5.3315    -0.7789
 1   3   4              1.4262     5.0890    -2.4752
 1   3  C2               1.4463     4.3064    -0.7428
 1   3  C3               1.4995     3.9883     0.9457
 1   3  H1               1.0935     5.3544    -0.6048
 1   4  C2               1.4666     4.6409    -0.6953
 1   4  C3               1.4976     6.5450     1.2073
 1   4  H1               1.0933     5.4381    -0.5778
 2   2   1   3          38.9045     1.4600
 2   2   1   4          62.8828     5.3921
 2   3   1   4          31.1956     1.7451
 2   1   2   3          72.7904     0.8498
 2   1   2  C3          113.7095     1.3544
 2   1   2  C2          114.0944     2.0150
 2   1   2  H1          109.1846     0.5439
 2   3   2  C3          126.5077     0.8136
 2   3   2  C2          124.2861     0.9957
 2   3   2  H1          119.3245     0.6145
 2  H1   2  C3           118.4643     0.4969
 2  H1   2  C2           113.9745     0.1840
 2  H1   2  H1           116.4148     0.4719
 2   1   3   2          71.3746     0.1737
 2   1   3   4          97.7602     0.5804
 2   1   3  C2          117.8560     1.0981
```

```
 2   1   3  C3          115.2384     2.5867
 2   1   3  H1          110.9192     0.4613
 2   2   3   4          122.3644     0.8526
 2   2   3  C2          124.5112     2.4193
 2   2   3  C3          123.6798     0.5464
 2   2   3  H1          116.5563     0.1281
 2   4   3  C2          118.9280     1.8938
 2   4   3  C3          120.9317     0.7171
 2   4   3  H1          117.1217     0.8904
 2   1   4   3           49.6771     0.1102
 2   1   4  C2          105.3175     0.1034
 2   1   4  C3          101.7406     0.1405
 2   1   4  H1           86.1229     0.1329
 2   3   4  C2          119.5362     0.3224
 2   3   4  C3          120.2062     0.4808
 2   3   4  H1          119.0980     0.5599
 2  H1   4  H1          113.9015     0.4620
 2  C2   4  H1          110.8934     0.3053
 2  C3   4  H1          116.0372     0.2697
 4  00   1   2  00        0.0000     0.0000     0.0000
 4   2   1   3  00        0.0000     0.0000     0.0000
 4   4   1   3  00        0.0000     0.0000     0.0000
 4  00   1   4  00        0.0000     0.0000     0.0000
 4  00   2   3  00        0.0000     0.0000     0.0000
 4  H1   2   3   4        0.0000     0.8902     0.0000
 4  C0   2   3   4        0.0000     1.7993     0.0000
 4  H1   2   3  H1        0.0000     0.0000     0.0000
 4  C0   2   3  H1        0.0000     0.0000     0.0000
 4  H1   2   3  C0        0.0000     0.0000     0.0000
 4  C0   2   3  C0        0.0000     0.0000     0.0000
 4  00   2  C2  00        0.0000     0.0000     0.0000
 4  00   2  C3  00        0.0000     0.0000     0.0000
 4  00   3   4  00        0.0000     0.0000     0.0000
 4   2   3   4  C2        0.0000    -0.1061     0.0000
 4   2   3   4  C3        0.0000     0.0000     1.4557
 4   2   3   4  H1        0.0000     4.0945     0.0000
 4   1   3   4  C2        0.0000     0.8770    -0.9674
 4   1   3   4  C3        0.0000     1.1033     0.0000
 4   1   3   4  H1        0.0000     0.0000    -0.8015
 4  00   3   4   1        0.0000     0.0000     0.0000
 4  00   3  C0  00        0.0000     0.0000     0.0000
 4   2   3  C2  C2        0.0000     0.0000     0.0000
 4   4   3  C2  C2        0.0000     0.0000     0.0000
 4  00   4  C3  00        0.0000     0.0000     0.0000
 4  00   4  C2  00        0.0000     0.0000     0.0000
 4   1   4  C2  00        0.0000     0.0000     0.0000
 5   4   3  00  00        0.0000     0.0000
-3
 C  PdTS_PP OPT
 9  Pd(-C0-C0(-1)-C0(.1)[.NX])(.P3)
-2
```

```
 1   1   6               2.3580     1.5684    -2.8961
 1   6  O3               1.6129     4.3412     1.9690
 1   6  C3               1.8392     3.6319    -0.4021
 1   6  C2               1.8390     3.2709    -1.3846
 1   6  H1               1.4138     3.5396     0.0321
 2   2   1   6          95.7465     0.1001
 2   3   1   6         149.9115     0.1005
 2   4   1   6         155.2452     0.1808
 2   1   6  H1         118.8123     0.1110
 2   1   6  C2         114.6107     0.2658
 2   1   6  C3         103.8950     4.7535
 2   1   6  O3         115.5134     1.1374
 2  H1   6  H1          98.7196     0.6170
 2  C2   6  C2         106.4260     2.7447
 2  C2   6  C3         103.5000     3.2797
 2   6  C2  N2         123.2000     0.5056
 2   6  O3  C3         125.0000     0.5000
 2   6  O3  C2         119.1945     0.2148
 4  00   1   6  00       0.0000     0.0000     0.0000
 4   6   1   2   3       0.0000     0.0000     0.0000
 4   6   1   3  00       0.0000     0.0000     0.0000
 4   4   3   1   6       0.0000     0.0000     0.9556
 4   2   3   1   6       0.0000     1.9210     0.0000
 4  H1   3   1   6       0.0000     0.0000     0.0000
 4   1   3   2   6       0.0000     0.0000     0.0000
 4   1   6  C2  C2       0.0000     0.0000     0.0000
 4   1   6  C2  N2       0.0000     1.0050     0.0000
 4   1   6  C3  00       0.0000     0.4001     0.0000
 4   1   6  C3  H1       0.0000     0.0000     0.0000
 4   1   6  C3  C0       0.0000     0.0000    -1.3260
 4   1   6  O3  C2       0.0000     0.0000     3.0462
 4   1   6  O3  C3       0.0000     0.0000     1.4410
 4   6   1   3  C0       0.0000     0.0000     0.0000
 4   6  O3  C0  C0       0.0000     0.0000     0.0000
-3
 C  PdTS_PN OPT
 9  Pd(-C0-C0(-1)-C0(.1)[.NX])(.P3)(.N0)
-2
 1   1   7               2.2482     1.5540    -3.0665
 2   2   1   7         165.3555     0.9410
 2   3   1   7         125.5870     0.1109
 2   4   1   7         103.8732     0.7572
 2   6   1   7          90.5092     0.1109
 4   7   1   2   3       0.0000     0.0000     0.0000
 4   7   1   3  00       0.0000     0.0000     0.0000
 4   7   1   3   2       0.0000     4.2795     0.0000
 4   7   1   3   4       0.0000     0.0000     1.9278
 4  00   1   6  00       0.0000     0.0000     0.0000
 4  00   1   7  00       0.0000     0.0000     0.0000
 4   6   2   3   7       0.0000     1.4028     0.0000
-3
```

```
 C  PdTS_N3 ligand OPT
 9  Pd.N3
-2
 1   2  H3               1.0203     6.9649    -1.4080
 1   2  C3               1.4570     3.2351    -0.3741
 2   1   2  H3         105.6811     0.1031
 2   1   2  C3         114.6750     2.0636
 2  H3   2  H3         112.9797     0.1956
 2  C3   2  C3         113.2437     1.1221
 4   1   2  C3  00       0.0000     0.0000     0.0000
-3
 C  PdTS_N2 ligand OPT
 9  Pd.N2
-2
 1   2  C3               1.4765     2.9993    -1.5609
 1   2  C2               1.3283     6.8499    -2.7262
 2   1   2  C3         112.1510     0.3768
 2   1   2  C2         123.3410     0.9884
 2  C3   2  C2         107.1845     0.1380
 4   1   2  C3  00       0.0000     0.0000    -2.5216
 4   1   2  C2  C2       0.0000     2.2465     0.0000
 4   1   2  C2  C3       0.0000     0.0000    -1.0000
 4   1   2  C2  O3       0.0000     0.6973     0.0000
 4   2  C2  C2  C2       0.0000     0.0000     0.0000
-3
 C  PdTS_amine OPT
 9  Pd-C0-C0(-1)-C0(.1).NX
-2
 1   4   5               1.9668     1.9093    -2.8841
 1   5  H3               1.0195     7.0392    -1.4042
 1   5  C3               1.4280     3.2872    -0.2903
 2   1   4   5         154.9079     0.1015
 2   3   4   5         106.5349     0.9533
 2   5   4  H1          93.3978     0.6182
 2   5   4  C2          99.5955     0.1251
 2   5   4  C3          97.7010     1.2985
 2   4   5  H3         110.6874     0.1069
 2   4   5  C0         111.0857     1.3239
 4   2   3   4   5       0.0000     0.0000     0.0000
 4   5   4  00  00       0.0000     0.0000     0.0000
 4  00   4   5  00       0.0000     0.0000     0.0000
 4   4   5  C0  00       0.0000     0.0000    -0.1909
-3
 C  Palladium oxazoline OPT
 9  Pd.N2=C2-O3-C3-C3-2
-2
 1   1   2               2.2505     1.3186    -2.9333
 1   2   3               1.2712    13.0304    -3.2480
 1   2   6               1.4724     7.9650    -0.8126
 1   3   4               1.3235     3.4594     0.6316
 1   4   5               1.4373     6.6772    -1.2473
```

```
 1   2  C0                1.4017     3.4671     0.1520
 1   4  C0                1.5151     2.5941     0.4716
 1   4   5               1.4440     5.1648    -1.9752
 2   1   2   3          130.6086     0.5693
 2   1   2   6          121.7180     0.0001
 2   3   2   6          107.8071     0.8095
 2   2   3   4          116.3343     0.6847
 2   2   3  C2           126.4541     0.5377
 2   2   3  C3           128.9152     0.3205
 2   4   3  C2           118.5661     1.6642
 2   4   4  C3           118.5277     0.4449
 2   3   4   5          112.6583     0.4766
 2   4   5   6          104.1000     0.6197
 4   1   2   3   4        0.0000     2.8509     0.0000
 4   1   2   3  C2        0.0000     2.8959     0.0000
 4   1   2   3  C3        0.0000     4.8651     0.0000
 4   1   2   6  00        0.0000     0.0000     0.0000
 5   2  00  00  00        0.0000     0.0000     0.0000
-3
 C  PdAllyl Oxazole OPT
 9  N2=C2-O2-C2=C2-1
-2
 1  Pd   1               2.1362     2.0971    -3.7679
 1   1   2               1.2849     5.2132    -1.9406
 1   1   5               1.3748     2.3660    -1.3658
 1   2   3               1.3209     3.4162     0.7539
 1   2  C0                1.4017     3.4671     0.1520
 1   3   4               1.3649     3.4196    -0.6118
 1   4  C0                1.5151     2.5941     0.4716
 1   4   5               1.3629     5.2176     0.3371
 2  Pd   1   2          124.4931     0.6121
 2  Pd   1   5          125.2020     2.3231
 2   2   1   5          103.2033     3.3837
 2   1   2   3          117.9564     2.4061
 2   1   2  C2           130.4264     0.2869
 2   1   2  C3           129.6924     0.1347
 2   1   5   4          104.1390     1.2007
 2   1   5  C3           121.5451     2.7335
 2   3   2  C2           117.3913     1.8003
 2   3   2  C3           120.5687     1.0388
 2   2   3   4          107.6874     2.1853
 2   3   4   5          102.3753     0.5901
 4   2   3   4   5        0.0000     0.6316     0.0000
 4   1   2   3   4        0.0000     0.4870     0.0000
 4   2   3   4  00        0.0000     0.0000     0.0000
 4  00   2   3   4        0.0000     0.0000     0.0000
 4  Pd   1   5   4        0.0000     1.1408     0.0000
 4  Pd   1   5  C3        0.0000     0.5337     0.0000
 4  Pd   1   2  00        0.0000     0.0000     0.0000
 4  Pd   1   2  C0        0.0000     0.4468     0.0000
-3
```

Supplementary Methods:
Details of Force Field Parameterization

The added substructures needed to describe the TS of this reaction was broken into eight different substructure. The first substructure described the atoms around the core, Pd-(C_{allyl}-C_{allyl}-C_{allyl}).N_{amine}, of the reaction which describes any of the parameters between the allyl and the metal center. There were four substructures developed to describe the P, N ligands. One substructure was used to describe the parameters between the metal and allyl with the phosphorus atom wjile a separate substructure was developed to describe the parameters metal and allyl with a general nitrogen atom. There were separate substructures to distinguish interactions between a N_{sp3} and a N_{sp2}. There was an two additional substructures developed to described an oxzaoline and oxazole moiety. The last substructure was used to describe the amine section.

With all of the substructures added to the MM3*, initial parameters needed to be estimated. The bond dipoles were all initially set to zero. The bond force constants were set to 1.0, with the exception of the force constant to describe the reaction coordinate which was set to 0.2. The angle force constants were set to 0.5, and the torsional terms were all set to zero. The equilibrium bond and angle values were set to the average of the interaction in the training set structures.

The force field parameters were then optimized starting with the bond dipoles, followed by the bond and angle force constants, the equilibrium bond and angle values, and finally the torsional terms. The equilibrium bond and angle values were optimized by tethering to the average reference value from the DFT optimized training set. This ensures that the values don't deviate to unrealistic parameters during the parameterization process. Once all of the parameters have been optimized, various different data types calculated by DFT and MM were compared to see how well the added force field substructures could reproduce the structural informational and the Hessian matrix. The bond dipoles, bonds, angles, torsions, and diagonal eigenvalues were calculated from the MM optimized structures and compared to the DFT optimized structures

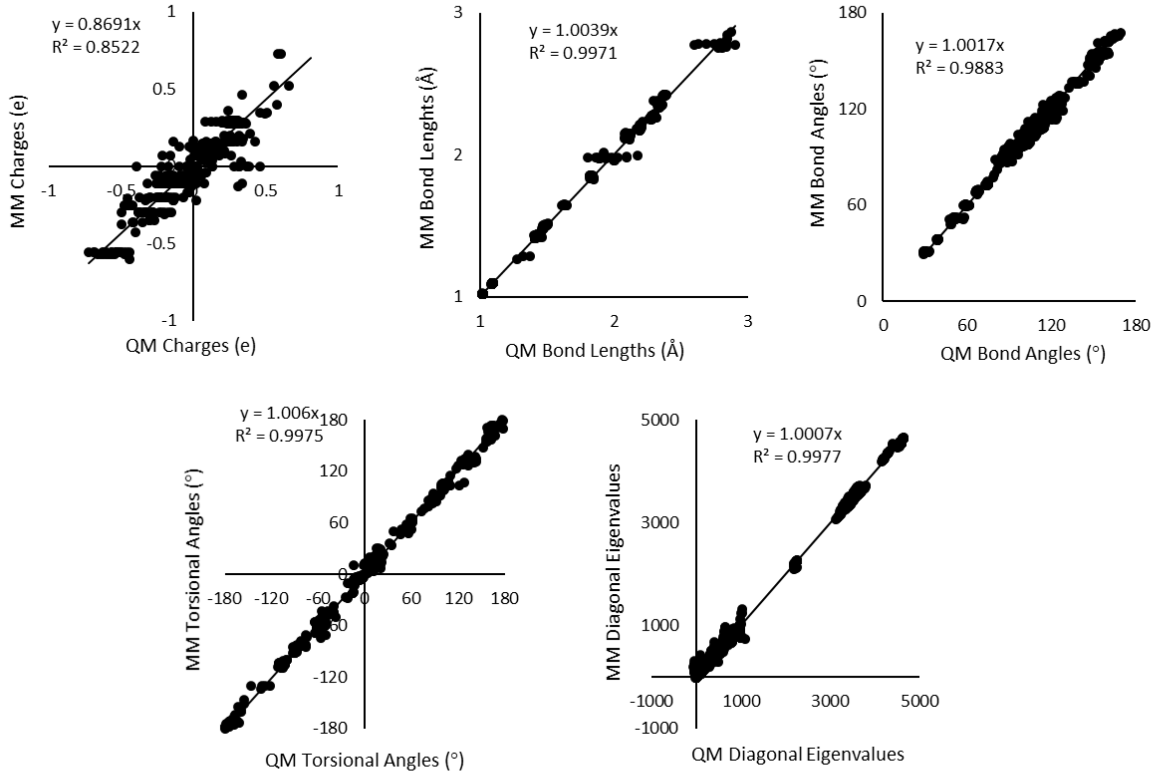

Supplementary Figure 2. Data comparison between the QM optimized data and the MM optimized data

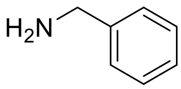

amine1

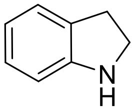

amine2

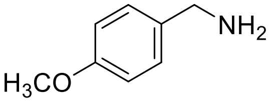

amine3

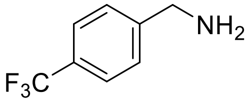

amine4

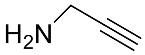

amine5

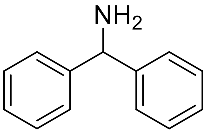

amine6

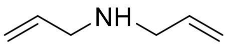

amine7

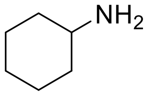

amine8

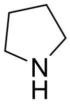

amine9

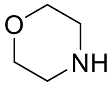

amine10

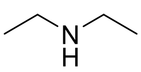

amine11

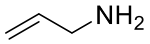

amine12

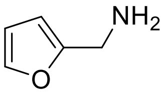

amine13

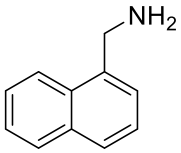

amine14

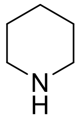

amine15

Supplementary Figure 3. Structures of the Nucleophiles in the Validation Set

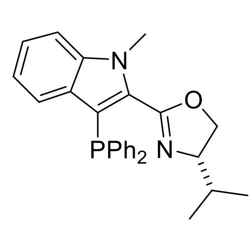

L1

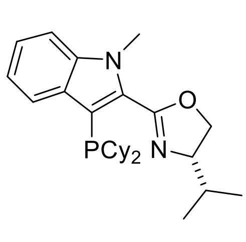

L2

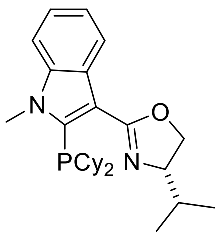

L3

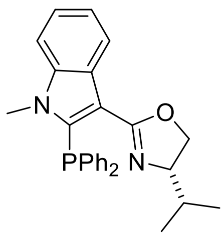

L4

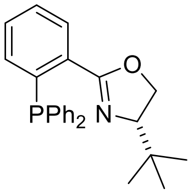

L5

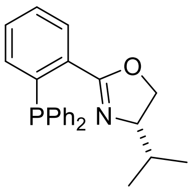

L6

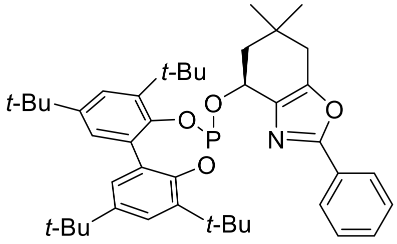

L7

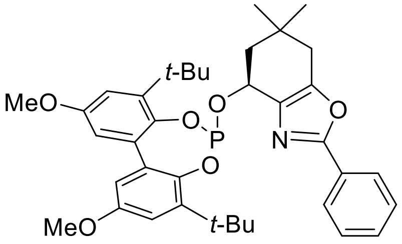

L8

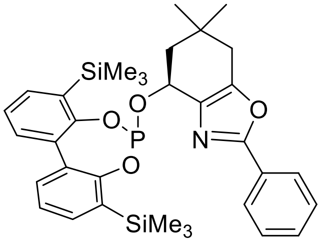

L9

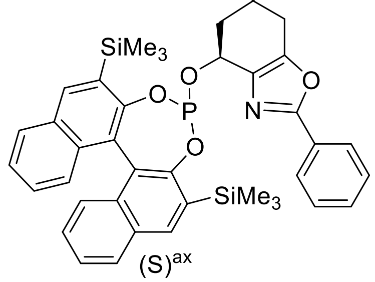

L10

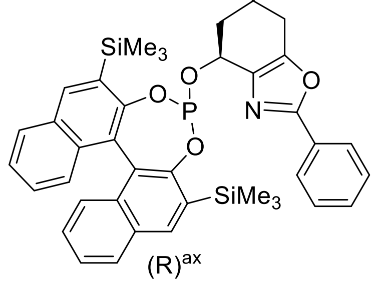

L11

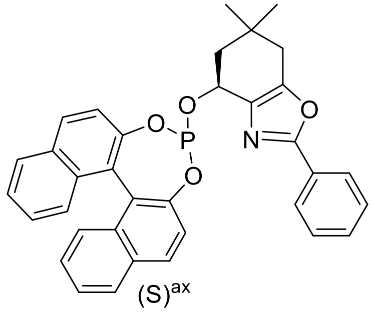

L12

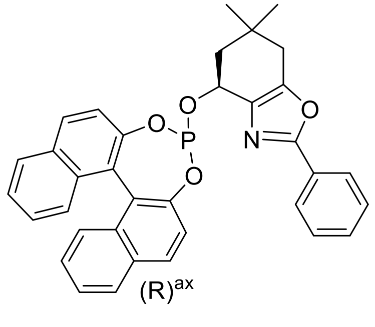

L13

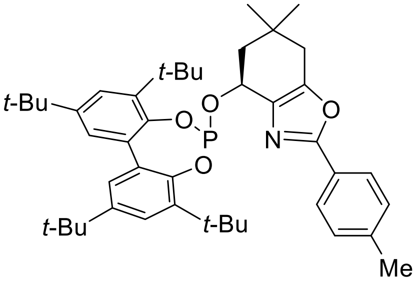

L14

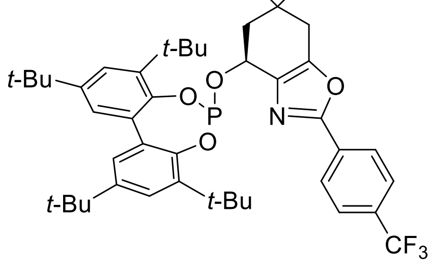

L15

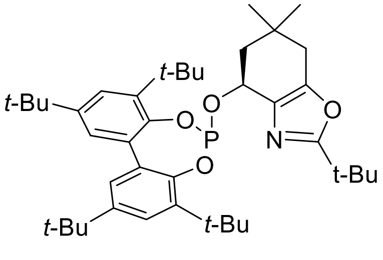

L16

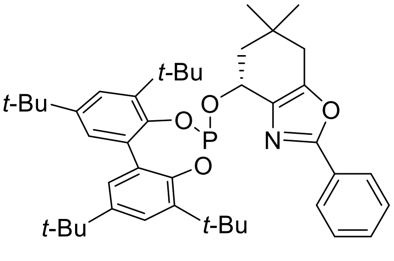

L17

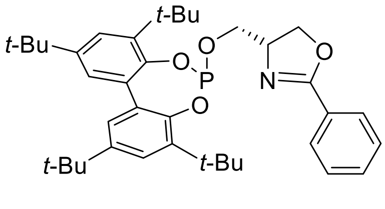

L18

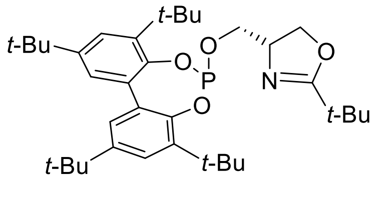

L19

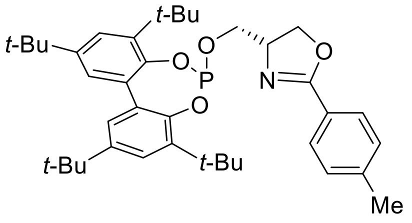

L20

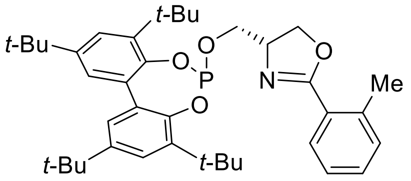

L21

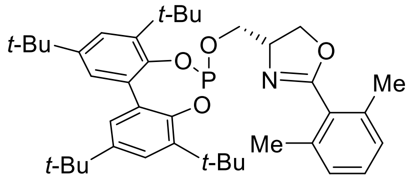

L22

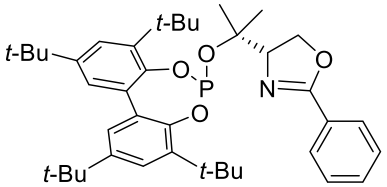

L23

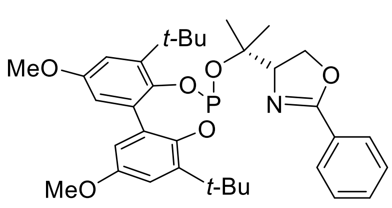

L24

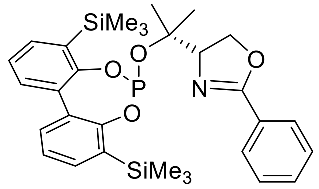

L25

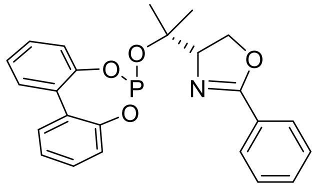

L26

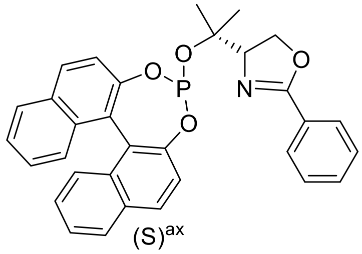

L27

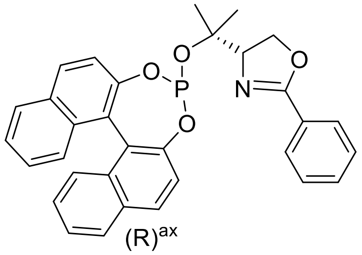

L28

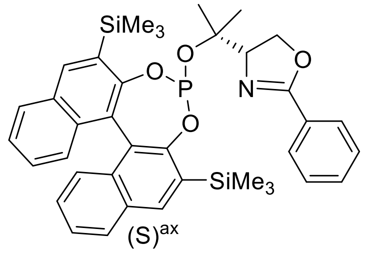

L29

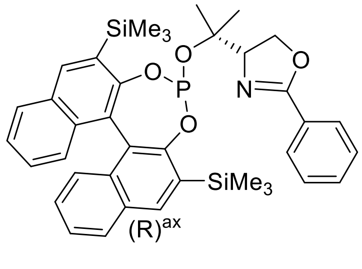

L30

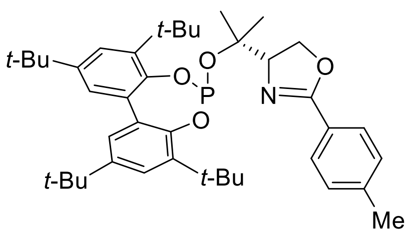

L31

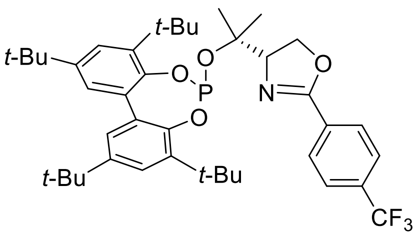

L32

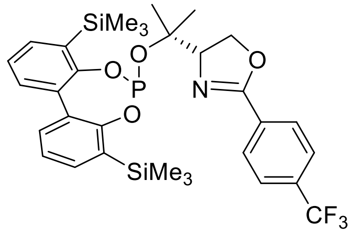

L33

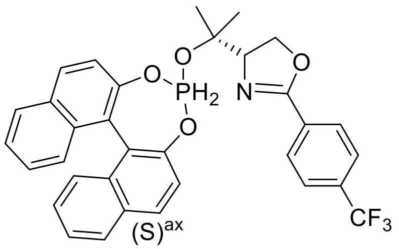

L34

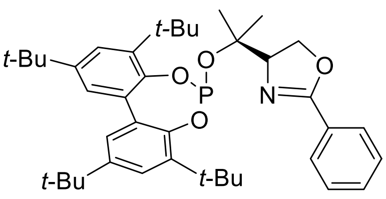

L35

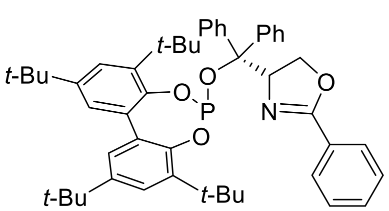

L36

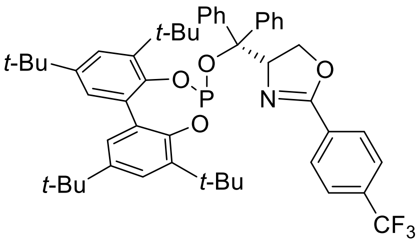

L37

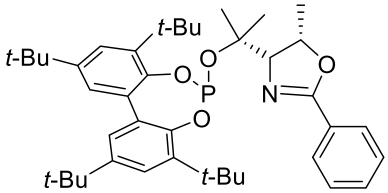

L38

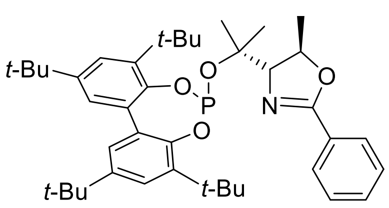

L39

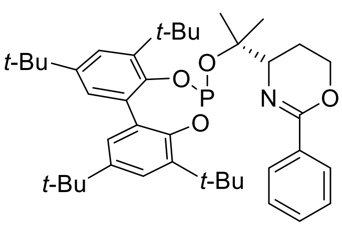

L40

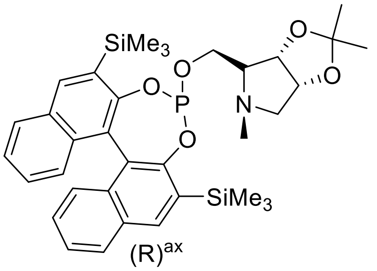

L41

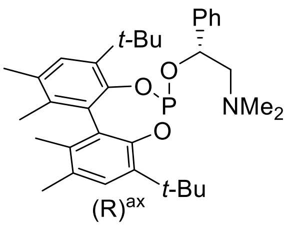

L42

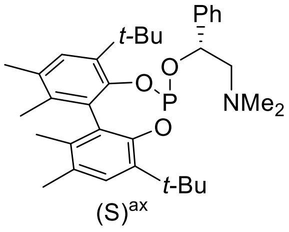

L43

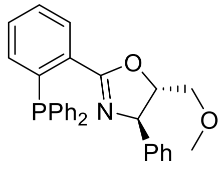

L44

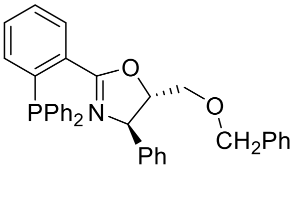

L45

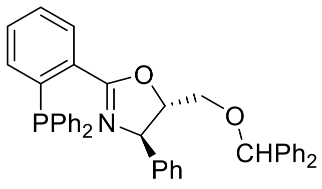

L46

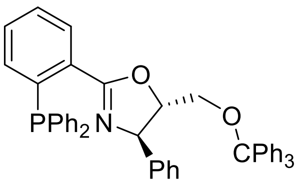

L47

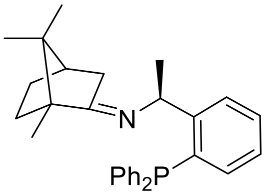

L48

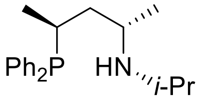

L49

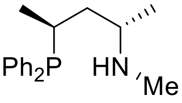

L50

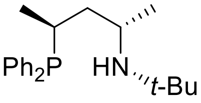

L51

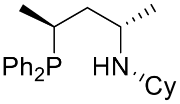

L52

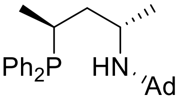

L53

Supplementary Figure 4. Structures of the Ligands in the Validation Set

Supplementary Table 3. Results for the Validation Set

	Nucleophile	Ligand	Abs. Conf (exp)	% ee (exp)	temp	$\Delta\Delta G^{\ddagger}$ (exp.)	$\Delta\Delta E^{\ddagger}$ (calc.)
Structure01	amine1	L1	R	-52	rt	-2.88	-8.76
Structure02	amine1	L2	R	-62	rt	-3.62	-18.96
Structure03	amine1	L3	R	-23	rt	-1.17	-18.96
Structure04	amine1	L4	R	-94	rt	-8.67	-5.96
Structure05	amine2	L5	S	95	rt	9.14	-18.96
Structure06	amine2	L6	S	86	rt	6.45	-8.59
Structure07	amine1	L7	R	-84	296	-6.01	5.10
Structure08	amine1	L8	R	-80	296	-5.41	2.75
Structure09	amine1	L9	R	-69	296	-4.17	2.67
Structure10	amine1	L10	R	-71	296	-4.37	6.29
Structure11	amine1	L11	R	-41	296	-2.14	3.61
Structure12	amine1	L12	R	-7	296	-0.35	4.88
Structure13	amine1	L13	S	5	296	0.25	5.98
Structure14	amine1	L14	R	-32	296	-1.63	1.35
Structure15	amine1	L15	R	-82	296	-5.69	6.62
Structure16	amine1	L16	R	-25	296	-1.26	6.93
Structure17	amine1	L17	S	84	296	6.01	-4.83
Structure18	amine1	L18	R	-55	rt	-3.08	-4.65
Structure19	amine1	L19	R	-9	rt	-0.45	-10.05
Structure20	amine1	L20	R	-50	rt	-2.74	-3.40
Structure21	amine1	L21	R	-32	rt	-1.65	-0.91
Structure22	amine1	L22	R	-5	rt	-0.25	-2.33
Structure23	amine1	L23	R	-87	rt	-6.65	-3.28

Structure24	amine1	L24	R	-86	rt	-6.45	-5.34
Structure25	amine1	L25	R	-92	rt	-7.93	-6.32
Structure26	amine1	L26	R	-92	rt	-7.93	-5.28
Structure27	amine1	L27	R	-93	rt	-8.27	-5.38
Structure28	amine1	L28	R	-91	rt	-7.62	-3.44
Structure29	amine1	L29	R	-57	rt	-3.23	-7.93
Structure30	amine1	L30	R	-90	rt	-7.34	-6.02
Structure31	amine1	L31	R	-83	rt	-5.93	-2.29
Structure32	amine1	L32	R	-93	rt	-8.27	-2.17
Structure33	amine1	L33	R	-96	rt	-9.71	-7.27
Structure34	amine1	L34	R	-84	rt	-6.09	-3.13
Structure35	amine1	L35	S	88	rt	6.86	3.34
Structure36	amine1	L36	R	-89	rt	-7.09	-8.71
Structure37	amine1	L37	R	-88	rt	-6.86	-8.27
Structure38	amine1	L38	R	-62	rt	-3.62	-0.31
Structure39	amine1	L39	R	-84	rt	-6.09	-2.77
Structure40	amine1	L40	S	8	rt	0.40	-8.09
Structure41	amine1	L41	S	91	273	6.93	5.62
Structure42	amine3	L41	S	90	273	6.68	5.48
Structure43	amine4	L41	S	91	273	6.93	4.42
Structure44	amine1	L42	S	97	296	10.30	2.37
Structure45	amine1	L43	R	-99	296	-13.03	-8.31
Structure46	amine1	L44	S	95	rt	9.14	10.12
Structure47	amine5	L44	S	94	rt	8.67	9.46
Structure48	amine6	L44	S	97	rt	10.44	13.20
Structure49	amine7	L44	s	96	rt	9.71	10.05
Structure50	amine8	L44	S	99	rt	13.20	9.71
Structure51	amine1	L45	S	84	rt	6.09	7.99
Structure52	amine1	L46	S	83	rt	5.93	4.63
Structure53	amine1	L47	S	88	rt	6.86	3.68
Structure54	amine1	L6	R	-96	313	-10.12	-8.51
Structure55	amine1	L48	S	99	rt	13.20	8.63
Structure56	amine9	L48	S	97	rt	10.44	11.34
Structure57	amine10	L48	S	86	rt	6.45	8.94
Structure58	amine11	L48	S	86	rt	6.45	9.71
Structure59	amine12	L48	S	98	rt	11.46	9.91
Structure60	amine13	L48	S	98	rt	11.46	10.61
Structure61	amine2	L48	S	87	rt	6.65	8.09
Structure62	amine4	L48	S	99	rt	13.20	9.71
Structure63	amine5	L48	S	97	rt	10.44	10.80
Structure64	amine14	L48	S	99	rt	13.20	12.54
Structure65	amine15	L48	S	98	rt	11.46	8.20
Structure66	amine7	L48	S	98	rt	11.46	11.87

Structure67	amine6	L48	S	94	rt	8.67	11.34
Structure68	amine1	L49	S	78	rt	5.21	9.64
Structure69	amine9	L49	S	61	rt	3.54	7.12
Structure70	amine12	L49	S	90	rt	7.34	10.05
Structure71	amine11	L49	S	82	rt	5.77	6.89
Structure72	amine16	L49	S	86	rt	6.45	5.45
Structure73	amine10	L49	S	80	rt	5.48	10.27
Structure74	amine16	L50	S	20	rt	1.01	0.24
Structure75	amine16	L51	S	54	rt	3.01	5.31
Structure76	amine16	L52	S	88	rt	6.86	7.29
Structure77	amine16	L53	S	62	rt	3.62	13.20

Supplementary Methods:

Details of DFT calculations to confirm lowest energy TS structure

To ensure that the TS being modeled is in fact producing the lowest energy structure, a few DFT calculations were performed using Structure 54 from the validation set as a model system (**Figure S6**). The three lowest conformations the conformational search results for the TS that leads to the R product and the TS that leads to the S product were optimized using DFT. The M06 functional form was used with a D3 empirical dispersion correction, and the basis sets used were LANL2DZ for palladium and 6-31+G*. These calculations were performed in the gas phase. A frequency analysis was also performed to confirm that the transition state structures contained one negative vibration corresponding to the formation of the carbon-nitrogen bond. The Gibbs free energy was obtain for each of the TS structures. The energy difference was then calculated between the lowest DFT optimized conformation of the TS that leads to the R product and the TS that leads to the S product. For Structure 54 the computed $\Delta G^{\ddagger}(R)–\Delta G^{\ddagger}(S)$ was computed to be 14.7 kJ/mol, and an HF energy of 11.4 kJ/mol.

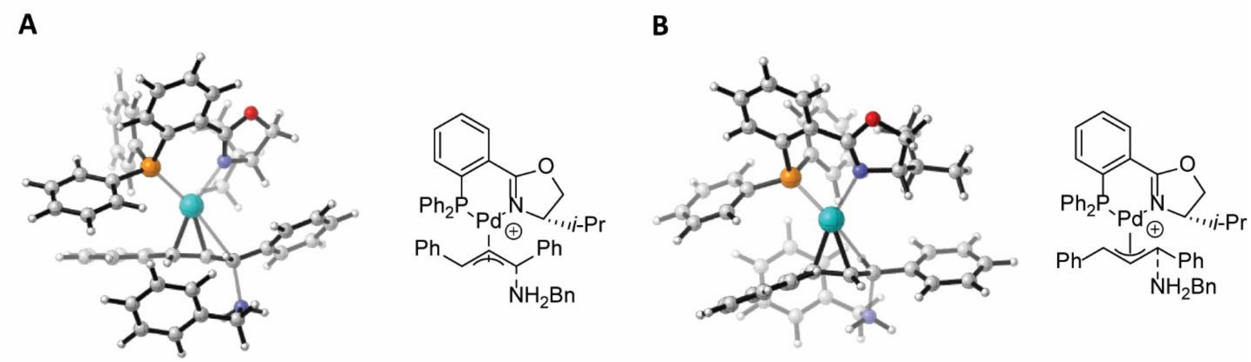

Supplementary Figure 6. Structure 54 used as the model system for the DFT calculations for A.) the TS that leads to the R product and B.) theTS that leads to the S product.

A single point of the DFT optimized structure were run using the LANL2TZ(f) basis set for palladium. The energy difference was calculated between the lowest energy conformation of the TS that leads to the R and S product. The HF energy was 11.6 kJ/mol which was comparable to the HF energy calculated using LANL2DZ as the basis set for palladium.

Calculations were also run to calculate the energy difference between the TS structures based upon how the ligand is coordinated. In Structure 54, the nucleophile is only modeled trans to the phosphorus atom. The lowest energy conformation for Structure 54 (in this case was the TS that leads to the R product) was re-optimized to flip the coordination of the ligand so the nucleophile was trans to the nitrogen atom. The energy difference was calculated between the two structures with the Gibbs free energy difference being 6 kJ/mol with the nucleophile being trans the ligand being lower in energy.

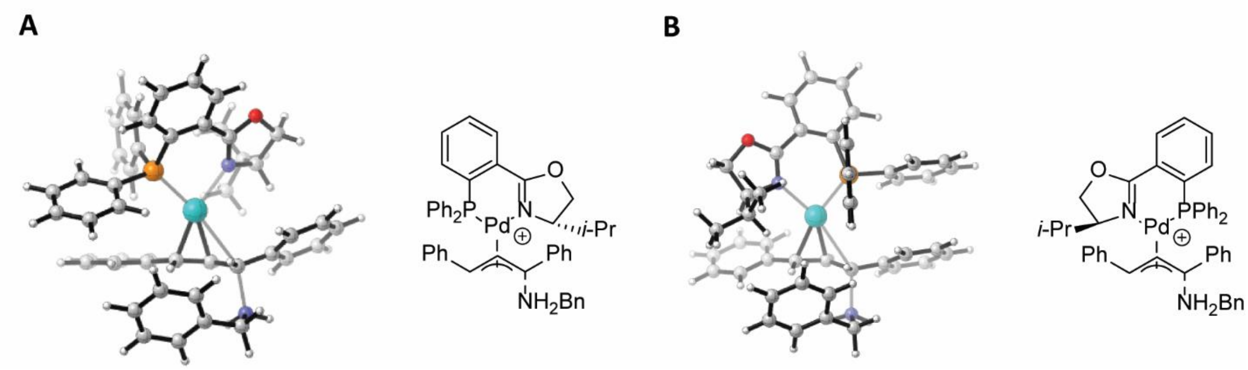

Supplementary Figure 7. Structures that were optimized to compare the energy difference between the two ways the ligand coordinate. A.) Structure 54 will the nucleophile trans to the P atom. B.) Nucleophile in trans to the N atom.

Lastly calculations were performed to consider the different conformations of the allyl. In Structure 54 only the syn/syn conformation is modeled. A conformational search was performed for the TS that leads to the R product of Structure 54 when the allyl is in the anti/anti conformation and syn/anti conformation. The three lowest energy conformers were optimized with DFT methods, and the lowest energy conformation compared to the lowest energy conformer of Structure 54. The Gibbs free energy difference was computed to be 55.6 kJ/mol between the syn/syn and anti/anti allyl conformations and 30.8 kJ/mol between the syn/syn and syn/anti allyl confromations. In each case the syn/syn conformation is significantly lower in energy showing that it is the preferred allyl conformation for the 1,3-diphenyl propenyl.

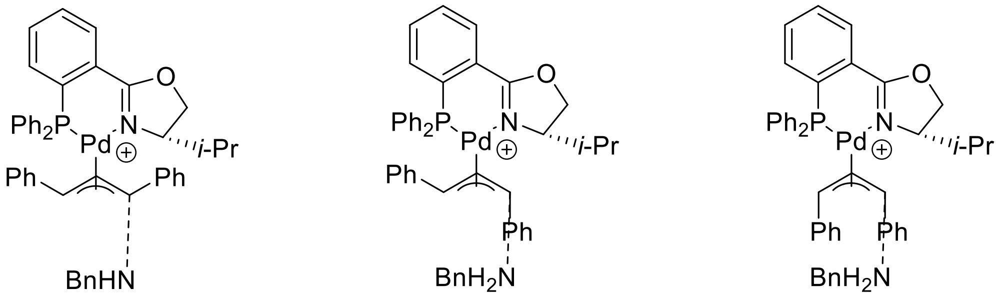

syn/syn syn/anti anti/anti

Supplementary Figure 8. Different conformations of the allyl

Supplementary Table 5. Coordinates for the DFT optimized structures used to confirm lowest energy of Structure 54.

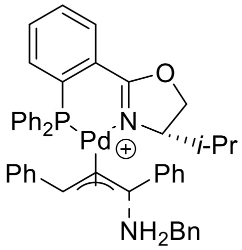

Structure 54 leading to R-product.
Gibbs Free Energy: -2431.095681
HF energy with LANL2TD for Pd: -2431.8151611
HF energy with LANL2TZ(F) for Pd: -2431.8230121
Imaginary Frequency: -325.5729
Cartesian coordinates:

C	1.57041	1.20258	0.88522
H	1.85465	1.77061	-0.00864
C	2.26053	-0.02765	1.12128
C	3.06003	-0.6211	0.09099
H	2.88782	-0.19735	-0.90231
H	2.30259	-0.46654	2.12082
Pd	0.30204	-0.33834	0.20875
N	-0.56948	-2.33885	-0.27315
P	-1.76395	0.47373	-0.51183
H	5.45401	-0.71868	0.46612
N	4.82507	0.05144	0.22561
H	4.78414	0.69047	1.02403
C	5.28092	0.75875	-0.99713
C	-0.48739	3.57294	3.8018

C	-0.32783	4.02369	2.49211
C	0.37341	3.25634	1.56932
C	0.91957	2.01347	1.9268
C	0.76057	1.57709	3.25106
C	0.06776	2.34981	4.1774
H	-1.03523	4.17257	4.52673
H	-0.75537	4.97719	2.18404
H	0.48294	3.60692	0.54031
H	1.16955	0.61554	3.56355
H	-0.04289	1.99191	5.20046
C	3.6863	-4.8547	-0.0422
C	3.37488	-4.1478	-1.20169
C	3.1873	-2.76918	-1.14686
C	3.29824	-2.08498	0.06738
C	3.62095	-2.80118	1.22637
C	3.81212	-4.17784	1.17156
H	3.83813	-5.9319	-0.08323
H	3.27946	-4.67049	-2.15223
H	2.92177	-2.21489	-2.0495
H	3.71639	-2.28012	2.18114
H	4.06136	-4.7262	2.07851
H	6.342	1.02772	-0.88835
H	5.20426	0.04278	-1.82763
C	2.77876	4.2016	-1.5957
C	2.70435	3.12993	-2.48158
C	3.5246	2.01798	-2.30264
C	4.43272	1.97405	-1.24365
C	4.51816	3.06537	-0.37404
C	3.68994	4.16989	-0.54124
H	2.00741	3.15602	-3.3187
H	3.4585	1.17637	-2.99516
H	5.24262	3.05604	0.44377
H	3.75609	5.00835	0.14969
H	2.13011	5.06662	-1.72704
C	-0.18571	-3.54917	0.47354
C	-1.95455	4.92109	-1.74564
C	-1.00412	4.05869	-2.29429
C	-0.97459	2.7234	-1.91081
C	-1.91704	2.22656	-1.00152
C	-2.86997	3.09187	-0.45993
C	-2.88154	4.43721	-0.82686
H	-1.97196	5.96992	-2.03766
H	-0.28435	4.43462	-3.02143

H	-0.21245	2.05114	-2.31536
H	-3.60325	2.71968	0.25623
H	-3.62426	5.10685	-0.39588
C	-4.85306	-0.12951	2.86605
C	-3.59731	0.42269	3.12001
C	-2.69236	0.59452	2.07922
C	-3.04003	0.23067	0.77186
C	-4.30271	-0.31248	0.52033
C	-5.20352	-0.49552	1.56857
H	-5.55995	-0.27491	3.6814
H	-3.31587	0.71442	4.13136
H	-1.70334	1.01157	2.28244
H	-4.58628	-0.60261	-0.49229
H	-6.1835	-0.926	1.36843
C	-3.4479	-1.81232	-4.20159
C	-2.69445	-2.49973	-3.25783
C	-2.15594	-1.84909	-2.13972
C	-2.4002	-0.46533	-1.9625
C	-3.17049	0.20269	-2.91729
C	-3.68874	-0.45539	-4.03007
H	-3.84825	-2.34146	-5.06406
H	-2.51408	-3.5642	-3.38313
C	-1.3873	-2.69004	-1.20062
H	-3.3774	1.26478	-2.78739
H	-4.28499	0.09654	-4.75472
O	-1.58594	-4.01548	-1.34518
C	-0.67882	-4.673	-0.44173
H	0.91142	-3.56605	0.56788
H	-1.23381	-5.46373	0.07571
H	0.12159	-5.12495	-1.04193
C	-0.80083	-3.58138	1.88321
H	-0.55785	-4.58685	2.27323
C	-2.31691	-3.42219	1.86909
H	-2.72332	-3.54337	2.88149
H	-2.6034	-2.41817	1.52406
H	-2.81888	-4.15727	1.22468
C	-0.15973	-2.55409	2.80615
H	-0.58054	-2.62362	3.81819
H	0.92845	-2.70043	2.87893
H	-0.34125	-1.5309	2.43985

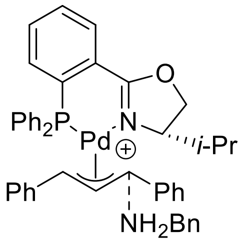

Structure 54 leading to S-product.
Gibbs Free Energy: -2431.090016
HF energy with LANL2TD for Pd: -2431.8107587
HF energy with LANL2TZ(F) for Pd: -2431.8185369
Imaginary Frequency: -331.2889
Cartesian coordinates:

C	-0.10315	-2.01064	-1.13515
H	0.11661	-2.46947	-0.1637
C	-1.47441	-1.67753	-1.3985
C	-2.3951	-1.73676	-0.2998
H	-1.92005	-1.6113	0.67617
H	-1.86254	-1.68546	-2.41938
Pd	-0.2377	0.06205	-0.86398
N	-0.75007	2.24086	-1.00717
P	1.58786	0.897	0.34237
H	-3.71044	-3.78195	-0.29372
N	-2.75701	-3.59007	0.02382
H	-2.10939	-4.07089	-0.6068
C	-2.55524	-4.03186	1.42891
C	2.76537	-3.06287	-4.13447
C	2.83989	-3.61147	-2.8566
C	1.89789	-3.26547	-1.89255
C	0.85784	-2.3736	-2.18795
C	0.79119	-1.83289	-3.48188
C	1.73573	-2.17208	-4.44236
H	3.50401	-3.32737	-4.88925
H	3.6381	-4.31013	-2.60729
H	1.9691	-3.67895	-0.88463
H	0.00558	-1.11712	-3.72816
H	1.67397	-1.73504	-5.43788
C	-6.40192	-0.24812	-0.33298
C	-5.66396	-0.26807	0.84841
C	-4.35342	-0.73386	0.83741
C	-3.76446	-1.18368	-0.35076
C	-4.51142	-1.15133	-1.53436
C	-5.82158	-0.68434	-1.5241
H	-7.42858	0.11377	-0.32816

H	-6.10863	0.08417	1.77781
H	-3.76579	-0.73347	1.75958
H	-4.06816	-1.49604	-2.46912
H	-6.3954	-0.66351	-2.44897
H	-3.20933	-3.41109	2.05667
H	-2.8906	-5.07428	1.53254
C	1.61225	-3.50515	2.34906
C	0.67227	-2.61561	2.86291
C	-0.68289	-2.80855	2.60413
C	-1.1123	-3.88289	1.82162
C	-0.16422	-4.79082	1.34025
C	1.18995	-4.60406	1.60221
H	0.99565	-1.76095	3.45757
H	-1.4178	-2.10973	3.01261
H	-0.48709	-5.65129	0.74924
H	1.91926	-5.31628	1.21846
H	2.67381	-3.34013	2.53247
C	-2.14503	2.69086	-1.12862
C	5.17112	-1.85058	1.32059
C	4.68271	-1.73337	0.01964
C	3.61031	-0.8888	-0.24834
C	3.02675	-0.13849	0.7806
C	3.5189	-0.26225	2.08413
C	4.58659	-1.11723	2.35135
H	6.00928	-2.51341	1.5308
H	5.13416	-2.30453	-0.79067
H	3.22058	-0.8119	-1.267
H	3.07177	0.31207	2.89656
H	4.96768	-1.2034	3.36822
C	-0.06405	2.30376	4.43383
C	-0.62306	1.21499	3.76648
C	-0.11834	0.82943	2.52915
C	0.95931	1.50974	1.95257
C	1.51035	2.6048	2.62493
C	0.99766	3.00022	3.85922
H	-0.46082	2.61578	5.39853
H	-1.46402	0.67605	4.20454
H	-0.5656	-0.00391	1.98179
H	2.34405	3.15421	2.18429
H	1.4309	3.85581	4.37474
C	3.44725	4.70085	-1.57969
C	2.07134	4.50862	-1.61975
C	1.47978	3.36835	-1.06144

C	2.30167	2.40586	-0.42602
C	3.68047	2.62071	-0.39181
C	4.25506	3.75299	-0.96534
H	3.88214	5.59154	-2.02862
H	1.43634	5.25041	-2.097
C	0.01228	3.26486	-1.17226
H	4.32392	1.88952	0.09714
H	5.3342	3.89066	-0.92305
O	-0.60724	4.4161	-1.49589
C	-1.99626	4.10294	-1.70562
H	-2.66115	2.02359	-1.8362
H	-2.58946	4.87174	-1.19913
H	-2.19075	4.15287	-2.7839
C	-2.86786	2.60531	0.22114
H	-2.75573	1.5539	0.54632
C	-4.35199	2.89417	0.03834
H	-4.89386	2.76424	0.98472
H	-4.52702	3.93004	-0.28996
H	-4.80897	2.22286	-0.7033
C	-2.25666	3.50207	1.28923
H	-2.76219	3.34423	2.25137
H	-1.18931	3.29285	1.44516
H	-2.36434	4.56878	1.04196

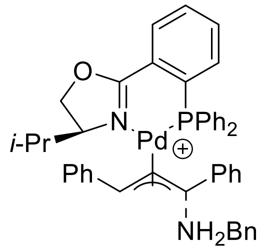

Structure 54 with nucleophile trans to N atom:
Gibbs Free Energy: -2431.093388
Imaginary Frequency: -360.2196
Cartesian coordinates:

C	2.12754	0.97368	-1.11728
H	2.5497	1.15037	-0.11848
C	1.01992	1.78761	-1.48126
C	0.40653	2.64715	-0.50294
H	0.57309	2.34403	0.53159
H	0.7688	1.94405	-2.5336
Pd	0.30116	-0.09733	-0.72783
H	1.13056	4.96714	-0.80495

N	1.5585	4.1794	-0.31294
H	2.41276	3.91623	-0.81124
C	1.8438	4.51509	1.10339
C	4.85742	-1.10047	-3.67283
C	5.25371	-0.65994	-2.41245
C	4.3586	0.03612	-1.60585
C	3.05094	0.30279	-2.03725
C	2.66629	-0.14448	-3.31078
C	3.56121	-0.83495	-4.11852
H	5.55272	-1.64782	-4.30682
H	6.26376	-0.85874	-2.0558
H	4.667	0.37876	-0.61506
H	1.6442	0.02019	-3.65502
H	3.2431	-1.18086	-5.10101
C	-3.33935	4.61453	-1.10781
C	-3.02903	4.05904	0.13035
C	-1.81205	3.41089	0.31391
C	-0.89892	3.29901	-0.73963
C	-1.22389	3.85313	-1.98403
C	-2.43527	4.51079	-2.16484
H	-4.29099	5.1236	-1.25284
H	-3.73999	4.11923	0.95274
H	-1.57569	2.96307	1.282
H	-0.52128	3.77741	-2.81585
H	-2.67713	4.9435	-3.13422
H	2.43491	5.44149	1.15095
H	0.87608	4.71464	1.5844
C	3.87372	1.17342	2.89418
C	2.54901	1.43974	3.23006
C	1.89909	2.53979	2.6762
C	2.56108	3.37683	1.77458
C	3.89428	3.10729	1.45291
C	4.54815	2.01048	2.00699
H	2.0203	0.78752	3.92476
H	0.86238	2.75404	2.94827
H	4.43184	3.76452	0.76519
H	5.58889	1.81361	1.75338
H	4.38562	0.31874	3.33396
N	0.78706	-2.22991	-0.31442
P	-1.84751	-0.74617	-0.00398
C	2.17718	-2.59964	-0.01215
C	-5.67837	1.7084	-0.80063
C	-4.72379	1.50878	-1.79757

C	-3.57055	0.78443	-1.51869
C	-3.37183	0.23143	-0.24839
C	-4.32721	0.44246	0.75036
C	-5.47551	1.18216	0.47311
H	-6.58041	2.27942	-1.01663
H	-4.87347	1.929	-2.79142
H	-2.81199	0.6412	-2.29212
H	-4.18172	0.02268	1.74653
H	-6.21835	1.3391	1.25402
C	-1.57453	-1.17414	4.60354
C	-0.96799	-0.10819	3.94103
C	-1.04565	-0.01736	2.55513
C	-1.75356	-0.9723	1.8155
C	-2.35904	-2.03795	2.48801
C	-2.26319	-2.14087	3.87472
H	-1.50657	-1.25341	5.68722
H	-0.42694	0.64877	4.50957
H	-0.53368	0.79229	2.02819
H	-2.90995	-2.7959	1.92909
H	-2.7339	-2.97825	4.38729
C	-3.08413	-5.00079	-1.43535
C	-1.75901	-4.74261	-1.10555
C	-1.34582	-3.46839	-0.69569
C	-2.30324	-2.43064	-0.59208
C	-3.62936	-2.7129	-0.92597
C	-4.02202	-3.98071	-1.34974
H	-3.37699	-5.99763	-1.75872
H	-1.02131	-5.53796	-1.17188
C	0.09144	-3.30757	-0.40595
H	-4.38105	-1.92819	-0.84867
H	-5.06438	-4.16509	-1.60417
O	0.76385	-4.4617	-0.24485
C	2.15898	-4.13157	-0.1131
H	2.82105	-2.16685	-0.79507
H	2.53833	-4.65181	0.77362
H	2.67723	-4.5136	-0.99988
C	2.62736	-2.02893	1.33704
H	2.48369	-0.93431	1.25979
C	4.11123	-2.30549	1.54395
H	4.46711	-1.86574	2.48524
H	4.32135	-3.38415	1.60311
H	4.71711	-1.89126	0.72441
C	1.78919	-2.52681	2.50635

H	2.09919	-2.03281	3.43841
H	0.72054	-2.31405	2.36254
H	1.90377	-3.60937	2.66764

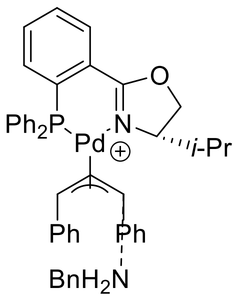

Structure 54 with allyl in anti/anti conformation:
Gibbs Free Energy: -2431.074273
Imaginary Frequency: -323.7381
Cartesian coordinates:

C	0.33976	1.92803	-1.72452
H	-0.18141	1.99585	-2.68889
C	1.53828	1.13819	-1.81811
C	2.54076	0.89785	-0.82914
H	3.10935	-0.01392	-1.03709
H	1.74524	0.66611	-2.78305
Pd	-0.14816	0.04424	-0.96227
N	-0.25266	-2.19204	-0.61164
P	-2.34307	-0.10351	-0.18128
H	4.1588	1.89869	-2.33646
N	4.10719	2.02886	-1.32452
H	3.84059	3.00138	-1.15877
C	5.39081	1.69253	-0.66623
C	-0.08249	5.62702	0.43677
C	0.70136	5.54761	-0.71408
C	0.81709	4.33854	-1.39512
C	0.17382	3.18245	-0.93083
C	-0.6192	3.28321	0.21247
C	-0.75095	4.49233	0.8898
H	-0.18095	6.57223	0.96812
H	1.21344	6.43298	-1.08895
H	1.41178	4.28346	-2.31022
H	-1.12666	2.39485	0.58485
H	-1.3793	4.54129	1.77874
C	2.36989	1.48575	3.41904
C	2.5456	0.21913	2.8651
C	2.61273	0.07404	1.48427

C	2.47115	1.17335	0.62987
C	2.32309	2.44268	1.19897
C	2.27151	2.59518	2.58147
H	2.32388	1.61091	4.49983
H	2.63997	-0.658	3.50603
H	2.77875	-0.91199	1.05002
H	2.23477	3.32592	0.56789
H	2.14538	3.59178	3.00193
H	6.20952	2.2639	-1.13093
H	5.31224	2.01703	0.37935
C	5.91057	-2.57081	-0.89176
C	5.808	-1.94527	0.3482
C	5.67024	-0.56069	0.42274
C	5.64033	0.21152	-0.73888
C	5.78075	-0.42098	-1.9783
C	5.90565	-1.80454	-2.05728
H	5.83568	-2.53558	1.26355
H	5.57025	-0.07447	1.39432
H	5.79636	0.17154	-2.89629
H	6.01133	-2.28552	-3.02826
H	6.01432	-3.65312	-0.95106
C	0.88547	-3.02386	-0.18457
C	-4.9653	3.6787	-0.50097
C	-4.31476	3.15704	-1.61992
C	-3.56247	1.99521	-1.50465
C	-3.47079	1.32838	-0.27592
C	-4.128	1.85241	0.84
C	-4.86993	3.0275	0.72505
H	-5.54323	4.59736	-0.58676
H	-4.38319	3.66647	-2.57969
H	-3.03022	1.60156	-2.37392
H	-4.05553	1.35269	1.80635
H	-5.37556	3.43282	1.60017
C	-2.34262	-1.25833	4.29574
C	-3.3966	-1.67602	3.48491
C	-3.40707	-1.34909	2.13128
C	-2.36051	-0.60308	1.5792
C	-1.29911	-0.19768	2.39629
C	-1.29404	-0.51994	3.75045
H	-2.33695	-1.5138	5.35425
H	-4.21437	-2.25755	3.90749
H	-4.23676	-1.6762	1.50221
H	-0.4638	0.36234	1.96591

H	-0.46385	-0.19786	4.37851
C	-4.62888	-3.5124	-2.37393
C	-3.29807	-3.69433	-2.01684
C	-2.57992	-2.68833	-1.35926
C	-3.22673	-1.47027	-1.03871
C	-4.5662	-1.31221	-1.39713
C	-5.26533	-2.31842	-2.06123
H	-5.16397	-4.30597	-2.89146
H	-2.79791	-4.63089	-2.24998
C	-1.18337	-2.99202	-1.00348
H	-5.08003	-0.38326	-1.15122
H	-6.30944	-2.16317	-2.32752
O	-0.85245	-4.29568	-1.07322
C	0.5632	-4.37483	-0.82371
H	1.81248	-2.59966	-0.60285
H	0.74095	-5.24476	-0.1831
H	1.06532	-4.5264	-1.78769
C	0.99455	-3.0373	1.34777
H	0.98611	-1.97727	1.65328
C	-0.18432	-3.70931	2.0399
H	-0.24299	-4.78462	1.81706
H	-0.08195	-3.61139	3.12873
H	-1.14301	-3.24742	1.7652
C	2.31625	-3.66298	1.77686
H	3.1805	-3.16556	1.30941
H	2.4407	-3.6045	2.86643
H	2.36745	-4.72889	1.50727

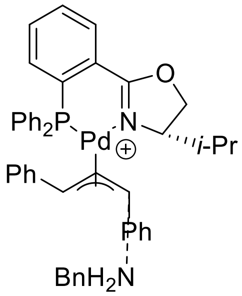

Structure 54 with allyl in syn/anti conformation:
Gibbs Free Energy: -2431.083812
Imaginary Frequency: -336.4797
Cartesian coordinates:

C	0.10949	-2.31835	-0.58519
H	0.22199	-2.6095	0.46302
C	-1.19541	-1.95823	-1.05063

C	-2.27784	-1.61975	-0.17871
H	-3.03093	-0.96179	-0.62226
H	-1.42811	-1.99583	-2.11757
Pd	0.0568	-0.21327	-0.5818
N	-0.38739	1.95219	-0.88908
P	2.07929	0.49686	0.3462
H	-3.65448	-3.26132	-1.18762
N	-3.51143	-3.11391	-0.18633
H	-3.01057	-3.93398	0.16172
C	-4.80311	-2.91815	0.51611
C	3.39837	-3.66	-2.9793
C	3.42775	-3.76958	-1.59031
C	2.33471	-3.36059	-0.8351
C	1.19377	-2.81856	-1.44622
C	1.17598	-2.7174	-2.84585
C	2.2647	-3.1376	-3.6024
H	4.2517	-3.98135	-3.57421
H	4.30692	-4.17382	-1.08976
H	2.36773	-3.43664	0.25391
H	0.3123	-2.28564	-3.35243
H	2.23081	-3.0511	-4.68779
C	-1.63423	-0.79353	3.98549
C	-2.05649	0.20834	3.1117
C	-2.29482	-0.0907	1.77538
C	-2.0835	-1.38304	1.28021
C	-1.67099	-2.38252	2.16599
C	-1.45346	-2.09078	3.51117
H	-1.45297	-0.56509	5.03431
H	-2.20231	1.22651	3.47065
H	-2.62976	0.69051	1.09229
H	-1.49864	-3.39973	1.80973
H	-1.13571	-2.88196	4.18889
H	-5.4681	-3.77025	0.30726
H	-4.58805	-2.91842	1.59197
C	-6.49597	0.84335	-0.69729
C	-6.09592	0.65178	0.62253
C	-5.56196	-0.5721	1.01932
C	-5.43186	-1.61678	0.1036
C	-5.85556	-1.42326	-1.21515
C	-6.37668	-0.19748	-1.61771
H	-6.19923	1.45734	1.34862
H	-5.23696	-0.7166	2.05081
H	-5.79116	-2.24168	-1.93642

H	-6.7039	-0.05778	-2.64697
H	-6.91427	1.80046	-1.00747
C	-1.25673	2.44893	-1.96812
C	5.4066	1.53154	-2.67112
C	4.51302	0.48843	-2.90825
C	3.49647	0.2249	-1.99633
C	3.3818	0.98964	-0.82945
C	4.27987	2.03553	-0.59512
C	5.28628	2.30705	-1.51903
H	6.19896	1.74368	-3.38727
H	4.60185	-0.12057	-3.80704
H	2.79036	-0.58719	-2.18523
H	4.19793	2.63797	0.3109
H	5.98238	3.12405	-1.33554
C	3.92729	-2.66246	3.15898
C	2.59884	-2.25575	3.30229
C	2.0779	-1.28084	2.46112
C	2.88305	-0.68595	1.48003
C	4.2112	-1.09359	1.34223
C	4.72859	-2.08221	2.18011
H	4.33491	-3.43345	3.81085
H	1.96629	-2.70493	4.06757
H	1.03239	-0.9756	2.56209
H	4.84438	-0.64982	0.57294
H	5.76441	-2.39774	2.06451
C	1.38391	4.27946	2.96681
C	0.73664	4.15628	1.74263
C	0.91256	3.01832	0.94631
C	1.78514	1.9933	1.37553
C	2.44121	2.14719	2.59882
C	2.23893	3.27172	3.39589
H	1.2233	5.16576	3.57751
H	0.0745	4.94659	1.39522
C	0.12693	2.97365	-0.30202
H	3.12867	1.37185	2.93683
H	2.76094	3.3597	4.34728
O	-0.1518	4.17557	-0.83856
C	-0.85147	3.92298	-2.07273
H	-1.01773	1.90283	-2.89259
H	-1.68667	4.62882	-2.13496
H	-0.15283	4.11985	-2.89467
C	-2.73207	2.20058	-1.62468
H	-2.80943	1.11711	-1.41681

C	-3.622	2.51189	-2.82081
H	-4.67081	2.27991	-2.59236
H	-3.57988	3.57686	-3.09435
H	-3.33382	1.92711	-3.70516
C	-3.1913	2.95909	-0.3847
H	-2.52528	2.79916	0.47644
H	-3.24708	4.04268	-0.56538
H	-4.1977	2.62959	-0.09169

Supplementary Methods:

Experimental

Pd-catalyzed amination of (*rac*)-1,3-diphenylallyl acetate with indoline using phosphine-oxazoline ligand L5.

A degassed solution of $[PdCl(\eta^3\text{-}C_3H_5)]_2$ (3.65 mg, 0.01 mmol) and **L5** (8.52 mg, 0.022 mmol) in dichloromethane (1 mL) was stirred for 30 min. Subsequently, a solution of the corresponding (*rac*)-1,3-diphenylallyl acetate (50.4 mg, 0.2 mmol) in dichloromethane (1 mL), indoline (27 μL, 0.24 mmol) and sodium carbonate (42.2 mg, 0.4 mmol) were added. The reaction mixture was stirred at room temperature for 18 hours. The reaction mixture was diluted with Et_2O (5 mL) and extracted with brine (3 x 10 mL) and the extract dried over $MgSO_4$. Solvent was removed and the product was purified by column chromatography (hexane/EtOAc 9:1).

Characterization of 1-(1,3-diphenylallyl)indoline.[1,2] ^{1}H NMR ($CDCl_3$, 401 MHz): δ 2.95–2.99 (m, 2H), 3.39–3.45 (m, 2H), 5.12 (d, *J* = 7.7 Hz, 1H), 6.36 (d, *J* = 7.9 Hz, 1H), 6.49 (dd, *J* = 15.9, 7.7 Hz, 1H), 6.61–6.68 (m, 2H), 6.95 (m, 1H), 7.08 (dd, *J* = 7.1, 1.4 Hz, 1H), 7.21–7.48 (m, 10H). ^{13}C NMR ($CDCl_3$, 100 MHz,): δ 28.4, 50.6, 64.1, 108.4, 117.5, 124.4, 126.5, 127.0, 127.3, 127.7, 127.8, 128.5, 130.5, 132.7, 136.7, 140.8, 151.3.

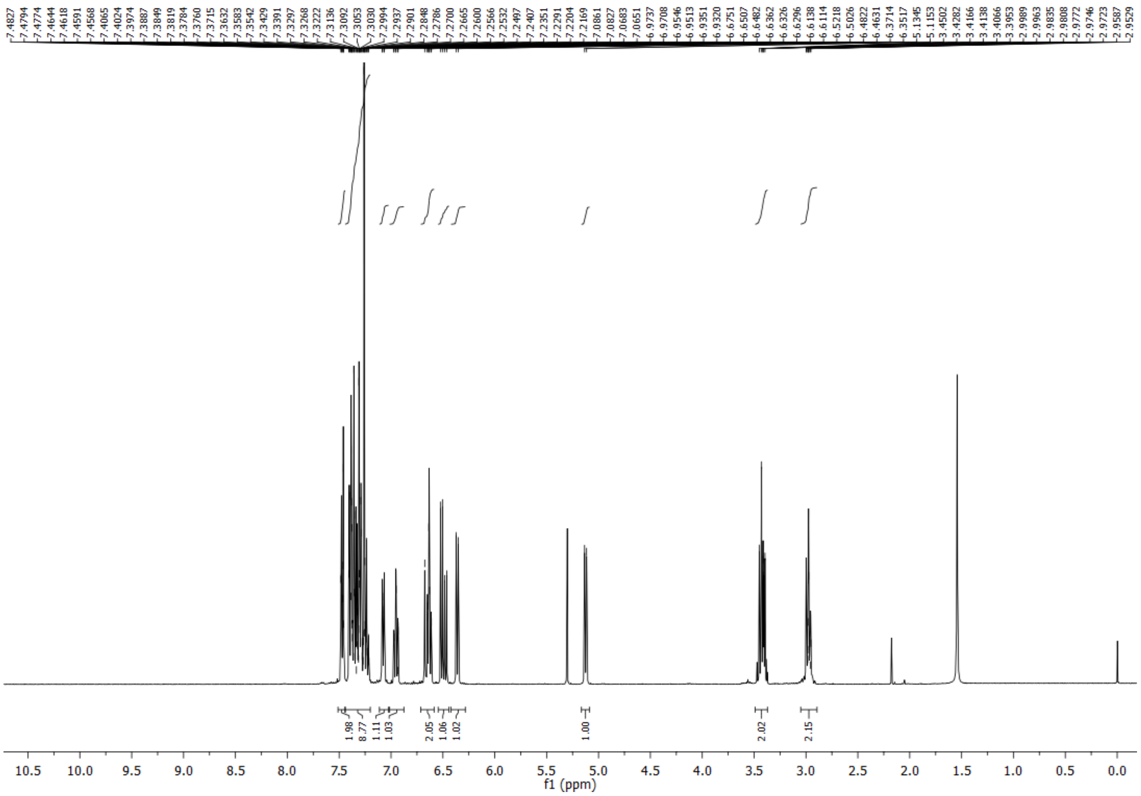

Supplementary Figure 9. ^{1}H NMR of 1-(1,3-diphenylallyl)indoline in $CDCl_3$.

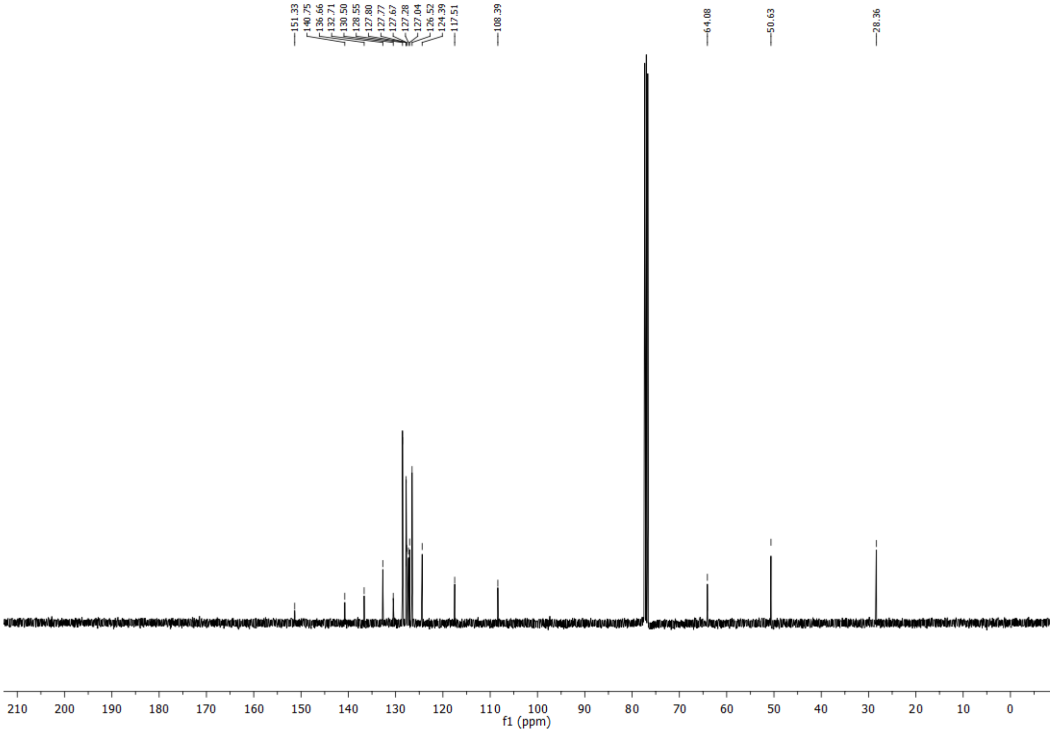

Supplementary Figure 10. $^{13}C\{^{1}H\}$ NMR of 1-(1,3-diphenylallyl)indoline in $CDCl_3$

Supplementary Methods:

Enantiomeric excess determination of 1-(1,3-diphenylallyl)indoline.[1,2] Enantiomeric excess was determined by HPLC using Chiralcel OD-H column (98% hexane/2-propanol, flow 0.5 mL/min). t_R 16.0 min (*S*, minor); t_R 17.2 min (*R*, major). The preferential formation of the (*R*) enantiomer was further confirmed by comparing the optical rotation of the sample $[\alpha]_D^{24}$: –6.8 (c 1.97 in $CDCl_3$) with those found in the literature $[\alpha]_D^{25}$: –10.8 (c 3.32 in $CDCl_3$), 86%(*R*) ee[1] and $[\alpha]_D^{23}$: +7.08 (c 2.36 in $CDCl_3$), 87%(*S*) ee[2].

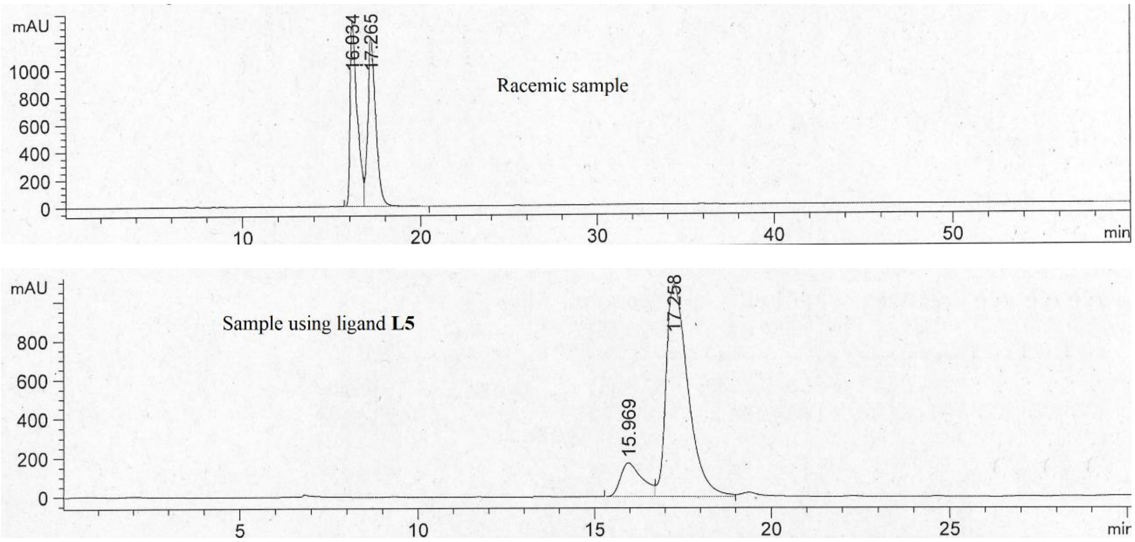

Supplementary Figure 11: Traces for chiral HPLC separation of 1-(1,3-diphenylallyl)indoline formed in a reaction catalyzed by **L5**

Supplementary Methods:

Experimental details for Pd-catalyzed amination of (*rac*)-1,3-diphenylallyl acetate with benzylamine using phosphite-oxazole ligands L7–L16.

Typical procedure. A degassed solution of [PdCl(η^3-C_3H_5)]$_2$ (0.9 mg, 0.0025 mmol) and the corresponding ligand (0.0055 mmol) in dichloromethane (0.5 mL) was stirred for 30 min. Subsequently, a solution of the corresponding (*rac*)-1,3-diphenylallyl acetate (0.5 mmol, 126.1 mg) in dichloromethane (1.5 mL) and benzylamine (131 µL, 1.5 mmol) were added. The reaction mixture was stirred at room temperature. After the desired reaction time, the reaction mixture was diluted with Et_2O (5 mL) and saturated NH_4Cl (aq) (25 mL) was added. The mixture was extracted with Et_2O (3 x 10 mL) and the extract dried over $MgSO_4$. Solvent was removed the product was purified by column chromatography (hexane/EtOAc 3:1). Enantiomeric excesses were measured by HPLC and the results are shown in Table S5

Enantiomeric excess determination of *N*-benzyl-1,3-diphenylprop-2-en-1-amine.[3] Enantiomeric excess was determined by HPLC using Chiralcel OD-H column (99% hexane/2-propanol, flow 0.5 mL/min). t_R 27.2 min (*R*); t_R 31.8 min (*S*), see Fig. S8. The preferential formation of the (*S*) enantiomer was further confirmed by comparing the optical rotation of the sample with 84% ee ($[\alpha]_D^{23}$: +15.4 (c 0.87 in $CDCl_3$)) with that found in the literature $[\alpha]_D^{23}$: +16.4 (c 0.85 in $CDCl_3$), 95%(*S*) ee.

Characterization of *N*-benzyl-1,3-diphenylprop-2-en-1-amine.[4] ^1H NMR ($CDCl_3$, 400 MHz), δ: 3.70 (m, 2H), 4.31 (dd, 1H, *J*= 7.6, 3.6 Hz), 6.24 (m, 1H), 6.49 (dd, 1H, *J*= 16, 3.6 Hz), 7.10-7.36 (m, 15H). ^{13}C NMR ($CDCl_3$), δ: 51.4, 64.6, 126.5, 127.0, 127.3, 127.4, 127.5, 128.2, 128.5, 128.6, 128.7, 130.5, 132.6, 137.0, 140.3, 142.8. HRMS (ESI+): m/z calcd. for $C_{22}H_{22}N$ $[M+H]^+$: 300.1747, found: 300.1746.

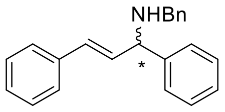

Supplementary Table 6. Enantiomeric excesses attained in the allylic amination using ligands **L7–L16**.

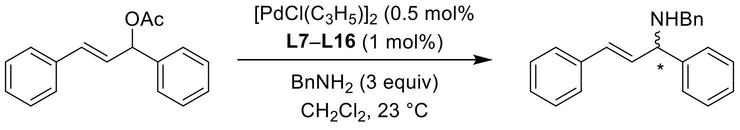

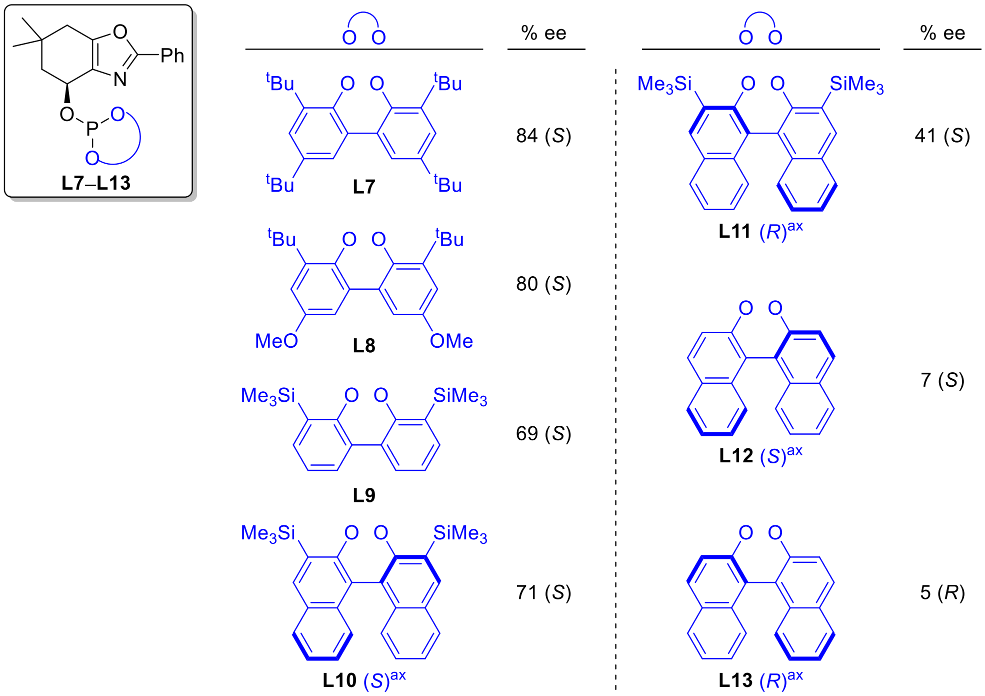

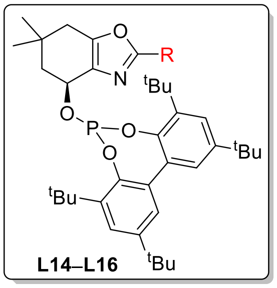

Ligand	R	% ee
**L14**	4-Me-C_6H_4	32 (*S*)
**L15**	4-CF_3-C_6H_4	82 (*S*)
**L16**	^{t}Bu	25 (*S*)

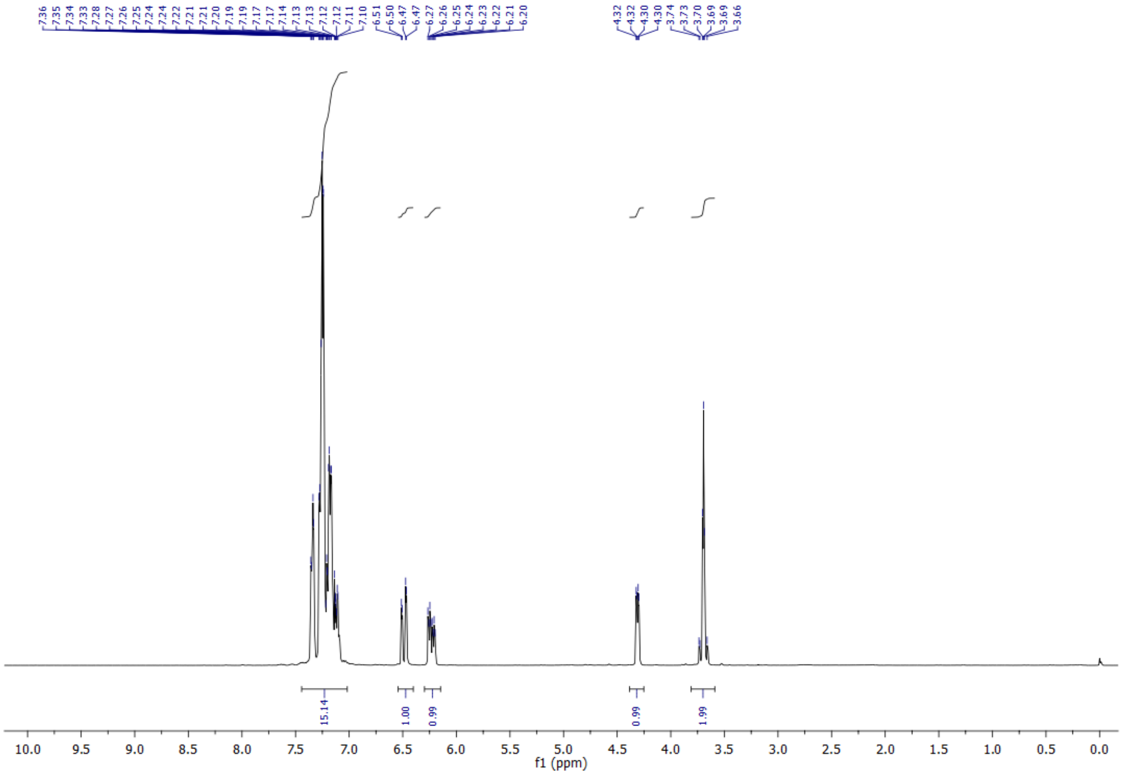

Supplementary Figure 12. ^{1}H NMR of *N*-benzyl-1,3-diphenylprop-2-en-1-amine in $CDCl_3$.

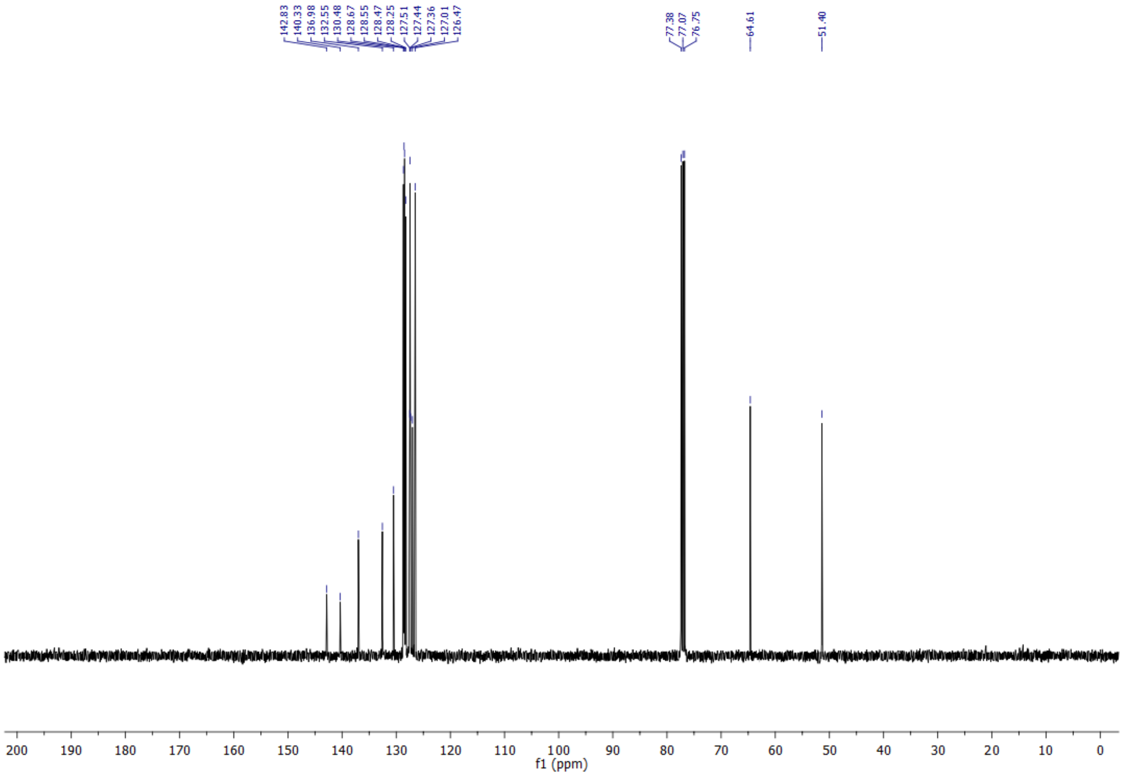

Supplementary Figure 13. $^{13}C\{^{1}H\}$ NMR of *N*-benzyl-1,3-diphenylprop-2-en-1-amine in $CDCl_3$

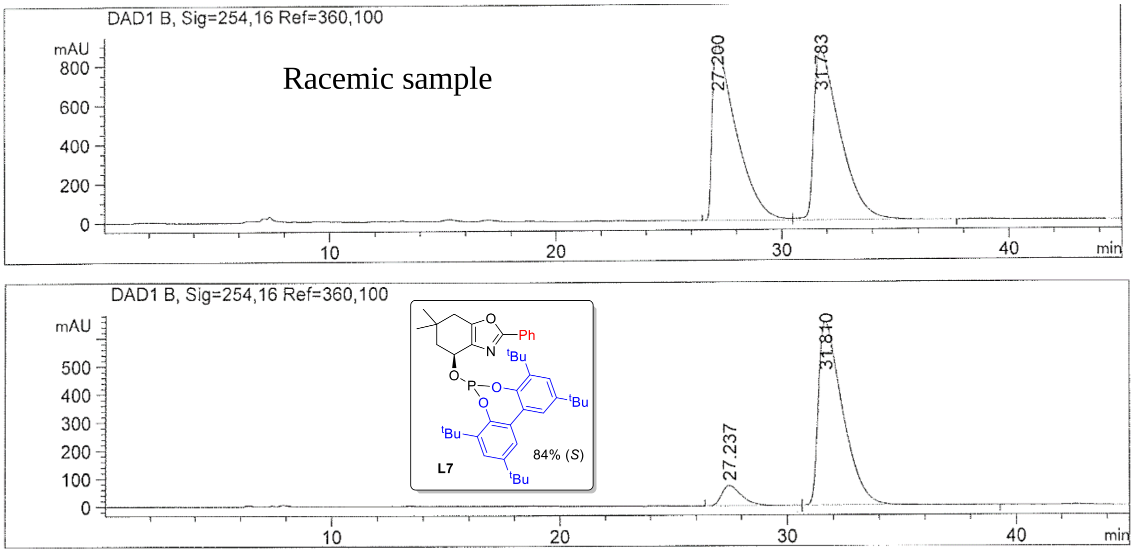

Supplementary Figure 14: Traces for chiral HPLC separation of *N*-benzyl-1,3-diphenylprop-2-en-1-amine formed in a reaction catalyzed by L7

Supplementary References

1. Nemoto, T.; Tamura, S.; Sakamoto, T. & Hamada, Y. Pd-catalyzed asymmetric allylic aminations with aromatic amine nucleophiles using chiral diaminophosphine oxides: DIAPHOXs. *Tetrahedron: Asymmetry* **19**, 1751–1759 (2008).
2. Liu, Q.-L.; Chen, W.; Jiang, Q.-Y.; Bai, X.-F.; Li, Z.; Xu, Z. & Xu, L.-W. A D-Camphor-Based Schiff Base as a Highly Efficient N,P Ligand for Enantioselective Palladium-Catalyzed Allylic Substitutions. *ChemCatChem* **8**, 1495–1499 (2016).
3. Popa, D. *et al.* Towards continuous flow, highly enantioselective allylic amination: ligand design, optimization and supporting. *Adv. Synth. Catal.* **351**, 1539–1556 (2009).
4. von Matt, P. *et al.* Enantioselective allylic amination with chiral (phosphino-oxazoline)pd catalysts. *Tetrahedron: Asymmetry* **5**, 573–584 (1994).
